# Protonation of Homocitrate and the E_1_ State of Fe-Nitrogenase Studied by QM/MM Calculations

**DOI:** 10.1021/acs.inorgchem.3c02329

**Published:** 2023-11-21

**Authors:** Hao Jiang, Kristoffer J. M. Lundgren, Ulf Ryde

**Affiliations:** Department of Computational Chemistry, Lund University, Chemical Centre, P.O. Box 124, Lund SE-221 00, Sweden

## Abstract

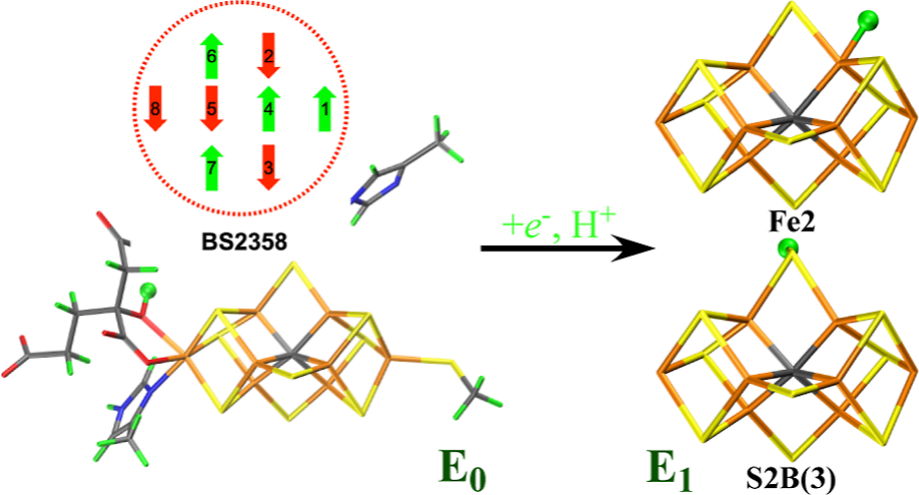

Nitrogenase is the only enzyme that can cleave the strong triple bond in N_2_, making nitrogen available for biological life. There are three isozymes of nitrogenase, differing in the composition of the active site, viz., Mo, V, and Fe-nitrogenase. Recently, the first crystal structure of Fe-nitrogenase was presented. We have performed the first combined quantum mechanical and molecular mechanical (QM/MM) study of Fe-nitrogenase. We show with QM/MM and quantum-refinement calculations that the homocitrate ligand is most likely protonated on the alcohol oxygen in the resting E_0_ state. The most stable broken-symmetry (BS) states are the same as for Mo-nitrogenase, i.e., the three Noodleman BS7-type states (with a surplus of β spin on the eighth Fe ion), which maximize the number of nearby antiferromagnetically coupled Fe–Fe pairs. For the E_1_ state, we find that protonation of the S2B μ_2_ belt sulfide ion is most favorable, 14–117 kJ/mol more stable than structures with a Fe-bound hydride ion (the best has a hydride ion on the Fe2 ion) calculated with four different density-functional theory methods. This is similar to what was found for Mo-nitrogenase, but it does not explain the recent EPR observation that the E_1_ state of Fe-nitrogenase should contain a photolyzable hydride ion. For the E_1_ state, many BS states are close in energy, and the preferred BS state differs depending on the position of the extra proton and which density functional is used.

## Abstract 2

We present the first QM/MM study of Fe-nitrogenase. We show that homocitrate is protonated on the alcohol atom and histidine on the NE2 atom. In the E_0_ state, Noodleman BS7-type broken-symmetry (BS) states are most stable (with β spin on the eighth Fe ion). For the E_1_ state, protonation of S2B is most favorable, 16–117 kJ/mol more stable than a hydride on Fe2. Many BS states are close in energy and sensitive to the protonation state.

## Introduction

Nitrogen is crucial in sustaining life on Earth, being a component of all amino acids and nucleic acids. Although N_2_ constitutes 78% of the Earth’s atmosphere, nitrogen remains a limiting factor for plant growth and is a main component in artificial fertilizers.^1^ The reason is that plants cannot metabolize N_2_ because it involves a strong and inert triple bond. The industrial conversion of nitrogen to ammonia occurs through the energy-intensive Haber–Bosch process, which involves high temperatures and pressures and accounts for almost 2% of the world’s total energy consumption.^2^

Nitrogenase (EC 1.18/19.6.1) is the only enzyme that can cleave the N–N bond in N_2_ and convert it to ammonia. It functions under ambient temperature and pressure. Nitrogenase exists in three forms: Mo-nitrogenase, V-nitrogenase, and Fe-only nitrogenase. Mo-nitrogenase is the most prevalent form, with the highest N_2_-reducing activity.^1,3−9^

**Figure 1:**
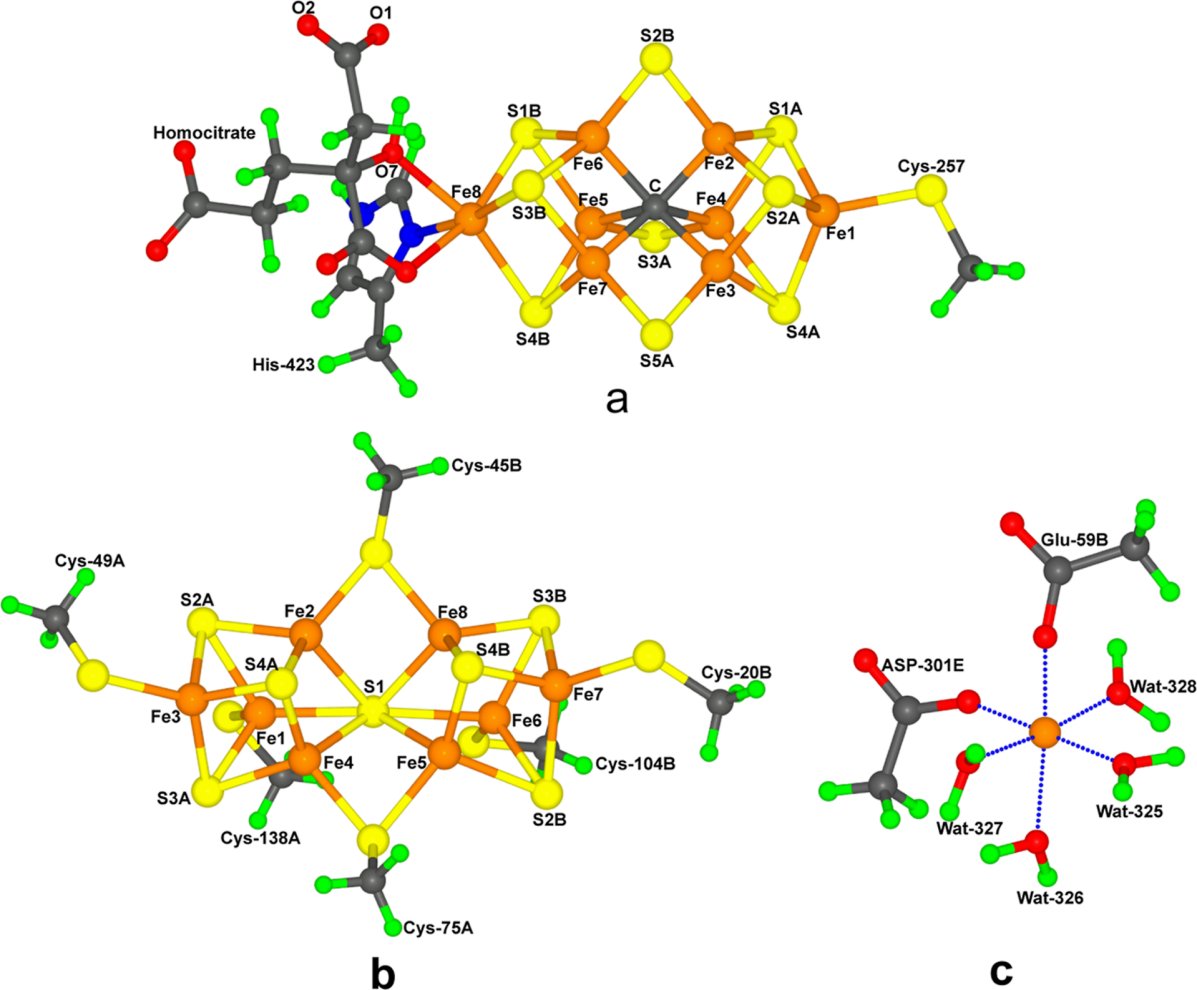
(a) FeFe cluster, (b) P cluster, and (c) Mg site in nitrogenase, also showing the atom names and the models used to calculate charges for the MM force field.

Crystal structures of Mo-nitrogenase have been known since 1992^10,11^ and of V-nitrogenase since 2017.^12^ However, the first crystal structure of Fe-only nitrogenase was published this year,^13^ and a cryogenic electron microscopy structure has also been presented.^14^ The studies have shown that all nitrogenases involve two proteins: the Fe protein and the Mo/V/FeFe protein. Electrons are supplied by the Fe protein, which also binds two ATP molecules. This binding triggers docking to the other protein and facilitates electron transfer. Hydrolysis of the ATP molecules induces the dissociation of Fe protein, thereby enabling further electron transfers. The MoFe protein is a α_2_β_2_ heterotetramer, whereas the VFe and FeFe proteins are α_2_β_2_γ_2_ heterohexamers, with one extra subunit. These proteins contain an Fe_8_S_7_Cys_6_ cluster called the P-cluster, which is used for electron transfer. In addition, they contain the active site, which is slightly different for the three types of nitrogenases. Mo-nitrogenase contains a catalytic MoFe_7_S_9_C(homocitrate) cluster, known as the FeMo cluster, V-nitrogenase contains a VFe_7_S_8_C(CO_3_)(homocitrate) cluster (FeV cluster), whereas Fe-only nitrogenase contains a Fe_8_S_9_C(homocitrate) cluster (the FeFe cluster, as shown in Figure 1a). In all three cases, the active-site cluster is coordinated to the protein via a cysteine and a histidine residue.^10,11,15−17^

The nitrogenases catalyze the reaction
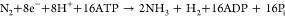
1although all three enzymes under normal N_2_ pressure produce more H_2_ and therefore consume more electrons, protons, and ATP molecules. The mechanism is commonly described by the Lowe–Thorneley scheme, which involves nine intermediates denoted as E_0_ to E_8_. These intermediates differ in the number of electrons and protons accumulated. Thorough biochemical, kinetic, and spectroscopic studies have demonstrated the necessity of reducing the E_0_ state to the E_4_ state before nitrogen binding can occur.^1,4−7,9,18^

It is generally believed that the three types of nitrogenases follow similar reaction mechanisms.^19,20^ However, recently, an EPR study of the one-electron reduced E_1_ state in Fe-nitrogenase (the E_1_ state is EPR active in this enzyme, in contrast to Mo-nitrogenase) suggested that it contains a Fe-bound hydride ion (based on the fact that the ligand is photolyzable) rather than a sulfur-bound proton.^21^ This is in contrast to Mo-nitrogenase, for which EXAFS measurements and combined quantum mechanical (QM) and molecular mechanical (MM) calculations have indicated that the E_1_ intermediate involves a protonated μ_2_ belt sulfide, probably S2B (atom names are shown in Figure 1a).^22^ This also agrees with previous QM and QM/MM studies, pointing out S2B as the energetically most favorable protonation site in the E_1_ state.^23,24^

Therefore, it is of great interest to examine whether there is an intrinsic difference in the protonation preferences of Mo- and Fe-nitrogenase in the E_1_ state. The recent crystal structure of Fe-nitrogenase makes such an investigation possible. In this article, we set up the first QM/MM calculations of Fe-nitrogenase, determining the proper protonation states of homocitrate and His-180, as well as the broken-symmetry (BS) state of the resting E_0_ state. Then, we evaluated the protonation preferences of the E_1_ state and discussed the implications of the findings.

## Methods

### Protein

All calculations were based on the recent crystal structure of Fe-only nitrogenase from *Azotobacter vinelandii* (PDB code 8BOQ), with a resolution of 1.55 Å.^13^ The calculations encompassed the entire α_2_β_2_γ_2_ heterohexamer as the subunits are intertwined. Likewise, two Mg^2+^ ions were retained because they are deeply buried in the protein, stabilizing the subunit interface. All crystal-water molecules were also kept, except eight that overlapped with each other or with protein atoms: HOH-223, 878, 976, 1086, 1122, 1173, 1255, and 1268.

The crystal structure is a mixture of the resting state (E_0_) and a turnover state in which the S2B ion is replaced by a light atom, modeled by O in the PDB file.^13^ The two states have almost equal occupancy (0.5/0.5 in subunit A and 0.4/0.6 in subunit D). Gln-176 also shows two conformations. In one (connected to the resting state), it points away from the FeFe cluster. In the other, it forms a hydrogen bond to His-180 (2.8 Å) and to the light atom, replacing S2B (2.5 Å). The OE1 atom in the resting-state conformation of Gln-176 is replaced by a stronger density, which is interpreted as the storage position of the replaced S2B. In our QM/MM calculations, we studied only the resting-state conformation so the extra O atom was deleted as well as the corresponding conformation of Gln-176 and the storage conformation of S2B.

The protonation states of all of the residues were determined through a thorough analysis of the hydrogen-bond pattern and the solvent accessibility. It was checked by the PROPKA^25^ and Maestro^26^ software. All Arg, Lys, Asp, and Glu residues were assumed to be charged, with the exceptions of Asp-78, Glu-62B, 245B, Lys-22, 55, 83, 339, 361, and 398B (residues without any letter after the residue number belong to subunit A, whereas those belonging to subunits B or C end with that letter; subunits D, E, and F were treated identically to subunits A, B, and C, respectively, and are not explicitly mentioned). Cys residues coordinating to Fe ions were considered deprotonated. A thorough manual investigation of all of the His residues gave the following protonation assignment: His-3, 4, 18, 248, 345, 364, 426, 452, 69B, 221B, and 41C were assumed to be protonated on the ND1 atom, His-181, 342, 140B, and 190B were presumed to be protonated on both the ND1 and NE2 atoms (and therefore positively charged), whereas the remaining 20 His residues were modeled with a proton on the NE2 atom. Furthermore, residue His-452D was flipped (i.e., the C and N atoms in the imidazole ring were exchanged). Protons were added by Maestro software, optimizing the hydrogen-bond network.

### Molecular Dynamics Simulations

All molecular dynamics (MD) simulations were performed with the Amber22 software.^27^ For the protein, we used the Amber ff14SB force field,^28^ and water molecules were described by the TIP3P model.^29^ For the metal sites, restrained electrostatic potential charges were employed, using electrostatic potentials calculated at the TPSS/def2-SV(P) level of theory^30,31^ and sampled with the Merz–Kollman scheme,^32^ although at a higher-than-default point density (∼2000/atom).^33^ The charge calculations were performed on a minimal model of the E_0_ resting state for the FeFe cluster, [Fe_8_S_7_(CH_3_S)_6_]^4–^ for the P-cluster (in the fully reduced state) and [Mg(CH_3_COO)_2_(H_2_O)_4_] for the Mg site, all shown in Figure 1. The charges are listed in Tables S1–S8. The metal sites were treated by a nonbonded model,^34^ and the positions of all heavy atoms were strongly restrained toward the crystal structure in the MD simulations (like all other heavy atoms in the protein; see below).

For the MD simulations, the protein was solvated in a periodic rectangular box of explicit water molecules, extending at least 10 Å from the solute using the leap program in the Amber suite. 234 Cl^–^ ions and 310 Na^+^ ions were added to neutralize the protein and obtain an ionic strength of 0.2 M.^35^ The ions were added by replacing random water molecules with the leap program in Amber software. However, some of them then end up inside the protein. This was avoided by using local software, ensuring that all of the counterions were in the solvent. The final system contained 251 429 atoms.

After the solvation, we performed 1000 cycles of minimization. This was followed by 1 ns constant-volume equilibration. Finally, the system was subjected to a 10 ns simulated annealing with a temperature of up to 373 K at constant pressure, followed by 1000 cycles of minimization. In all these calculations, the heavy atoms of the protein and the oxygen atoms of crystal-water molecules were restrained toward the crystal structure with a force constant of 10,000 kcal/mol/Å^2^.

The temperature was kept constant at 300 K using Langevin dynamics, with a collision frequency of 2 ps^–1^.^36^ The pressure was kept constant at 1 atm using Berendsen’s weak-coupling isotropic algorithm with a relaxation time of 1 ps.^37^ Long-range electrostatics were handled by particle-mesh Ewald summation^38^ with a fourth-order B spline interpolation and a tolerance of 10^–5^. The cutoff radius for Lennard-Jones interactions was set to 8 Å. All bonds involving hydrogen atoms were constrained to their equilibrium values using the SHAKE algorithm (except in the constant-volume simulations),^39^ allowing for a time step of 2 fs during the simulations. The final structure was used for QM/MM calculations.

In addition, we set up eight MD simulations of the protein in different protonation states of homocitrate, His-180, and the FeFe cluster. In these, no restraints toward the crystal structure were used in the final steps. For the metal sites, we used restraint for all metal–ligand bonds with the average distance in the two subunits of the crystal structure as the target (but for the FeFe cluster, distances from the QM/MM calculations with the various protonation states were used) and a force constant of 50 kcal/mol/Å^2^. This ensures that the metal sites are kept intact, with a structure close to the crystal structure, but it also allows for some dynamics and avoids problems with water molecules that are often encountered with a bonded potential.^34^ The Fe, S, and carbide ions of the FeFe and P-clusters were restrained to the crystal structure with a force constant of 1000 kcal/mol/Å^2^.

The same simulations were performed as for the equilibration of the crystal structure (but with a force constant of 1000 kcal/mol/Å^2^), except for the final two steps, which were replaced by a 1 ns equilibration, and a 100 ns production simulation in which no restraints were applied (except for the Fe, S and carbide ions). 1000 snapshots were collected during the production simulation.

### QM/MM Calculations

QM/MM calculations were performed with the ComQum software.^40,41^ In this approach, the protein and solvent were split into three subsystems: system 1 (the QM region) was relaxed by the QM methods. System 2 contained all residues or water molecules with any atom within 6 Å of any atom in system 1. It was optionally relaxed by a MM minimization in each cycle of the QM/MM optimization, using updated charges for the QM system. System 3 contained the remaining part of the protein and the solvent, and it was kept fixed at the original coordinates (equilibrated crystal structure to reduce the risk that different calculations end up at different local minima).

In the QM calculations, system 1 was represented by a wave function, whereas all the other atoms were represented by an array of partial point charges, one for each atom, taken from the MM setup. Thereby, the polarization of the QM system by the surroundings is included in a self-consistent manner (electrostatic embedding). When there is a bond between systems 1 and 2 (a junction), the hydrogen link-atom approach was employed: the QM system was capped with hydrogen atoms (hydrogen link atoms, HL), the positions of which are linearly related to the corresponding carbon atoms (carbon link atoms, CL) in the full system.^41,42^ All atoms were included in the point-charge model, except the CL atoms.^34^

The total QM/MM energy in ComQum was calculated as^40,41^
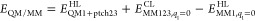
2where  is the QM energy of the QM system truncated by HL atoms and embedded in the set of point charges modeling systems 2 and 3 (but excluding the self-energy of the point charges).  is the MM energy of the QM system, still truncated by HL atoms but without any electrostatic interactions. Finally,  is the classical energy of all atoms with CL atoms and with the charges of the QM region set to zero (to avoid double-counting of the electrostatic interactions). Thus, ComQum employs a subtractive scheme with electrostatic embedding and van der Waals link-atom corrections.^43^ No cutoff is used for any of the interactions in the three energy terms in eq 2.

The QM calculations for QM/MM were performed using the Turbomole software (version 7.7).^44^ We employed four density functional theory (DFT) methods, TPSSh,^45^ r^2^SCAN,^46^ B3LYP,^47−49^ and TPSS.^31^ The former two were selected because they have been shown to give the best structures for nitrogenase models.^50^ B3LYP gave the best results in two recent calibration studies on simple nitrogenase model systems with one or two Fe ions,^51,52^ whereas TPSS has been used in most of our previous studies.^23,53,54^ The calculations involved either the def2-SV(P)^30^ basis set for all atoms or the def2-TZVP^30^ basis set for the FeFe cluster (including the added proton), homocitrate, Cys-257 and His-423, and the def2-SV(P) basis set for other groups. To enhance computational efficiency, Coulomb interactions were expanded in an auxiliary basis set by using the resolution-of-identity (RI) approximation.^55,56^ Empirical dispersion corrections were applied using DFT-D4,^57^ as implemented in Turbomole.

Two sizes of the QM system were used. In the minimal model, the FeFe cluster was represented by Fe_8_S_9_C(homocitrate)(CH_3_S)(methylimidazole) (56 atoms, see Figure 1a), where the last two groups model Cys-257 and His-423, taken from the A subunit of the protein (the two P clusters and the FeFe cluster in subunit D were modeled by MM in the fully reduced and the E_0_ resting states). In the large model, we added all groups that form steric or hydrogen-bond interactions with the FeFe cluster: Val-57, Lys-83, Gln-176, His-180, and Phe-362 (side chains), four water molecules, the whole Ser-260 (except the O atom, but including CH_3_CO– from the previous residue), as well as the backbone from Pro-335 to Lys-339, including the full side chain of the latter residue (187 atoms in total, as shown in Figure S1). Following experiment data,^58^ we used the oxidation state-assignment Fe_4_^III^ Fe_4_^II^ and a singlet spin state, *S* = 0^59^ for the resting E_0_ state and a doublet state for the E_1_ state.^21^

**Figure 2:**
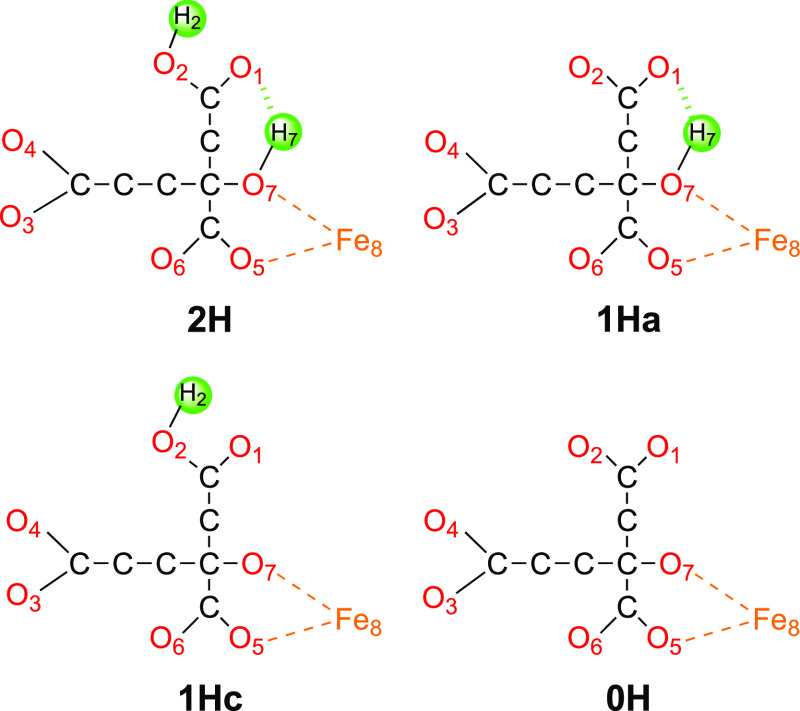
Four considered protonation states of homocitrate, 2H, 1Ha, 1Hc, and 0H. Atom numbers are also shown. Nonpolar H atoms are omitted. Charge of homocitrate is −2, −3, −3, and −4, respectively, in these four protonation states.

Four different protonation states of homocitrate were tested: fully deprotonated (0H; net charge −4), with one proton either on the alcohol (1Ha) or on the O2 carboxylate atom (1Hc; both with a −3 net charge), or with protons on both of these groups (2H; net charge −2). These protonation states are shown in Figure 2. They give net charges for the large QM region of −5 to −3.

**Figure 3:**
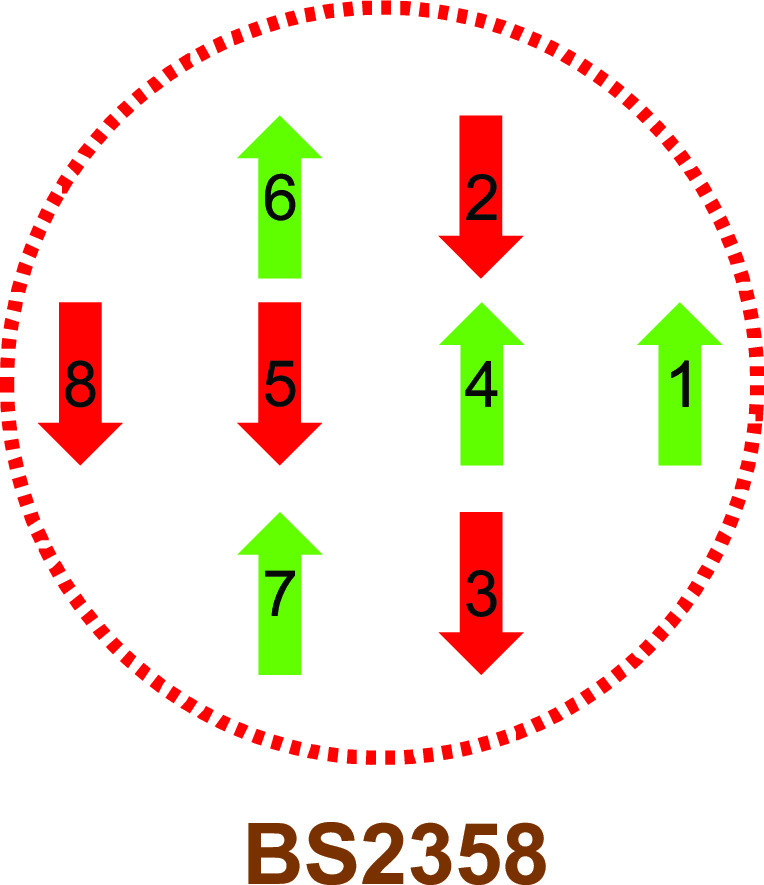
Energetically lowest BS state for the resting E_0_ state, showing the local spin surplus on each Fe ion.

The electronic structure in all QM calculations was described using the BS approach.^60^ Each of the eight Fe ions was modeled in the high-spin state, and these spins were then coupled antiferromagnetically to a singlet (E_0_) or doublet (E_1_) state. This means that the eight Fe ions should have either a surplus of α (four Fe ions) or β (four Fe ions) spin. Such a state can be selected in 70 different ways. The various BS states were obtained either by swapping the coordinates of the Fe ions^61^ or with the fragment approach by Szilagyi and Winslow.^62^ The various BS states are denoted simply by giving the number of the four Fe ions with β spin (Fe ion numbers are shown in Figure 1a), e.g., BS-2358, as shown in Figure 3.

### Quantum Refinement

In crystallographic refinement, the goal is to find the model (coordinates, *B*-factors, and occupancies) that best explains the observed structure factors. This is done by minimizing the difference between the experimentally obtained structure factors and those calculated from the current model. Due to the limited resolution obtained in protein crystallography, it is often necessary to supplement the refinement target function with empirical restraints that encode chemical knowledge. These restraints are obtained from either high-resolution crystallography of small molecules or quantum chemical calculations. In terms of computational chemistry, this is a MM force field. The refinement target then becomes a pseudoenergy function of the form
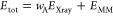
3where *E*_Xray_ is a crystallographic goodness-of-fit criterion (typically a least-squares or a likelihood function), *E*_MM_ is the empirical restraints, and *w*_A_(63) is a weight factor determining the relative importance of the two terms.

This approach works well when restraints of high accuracy are available, which is the case for amino acid residues and nucleic acids. However, for cofactors, substrates, inhibitors, and metal sites^34^ (which often are found in the most interesting part of the structure), less experimental information is available and the restraints are therefore less accurate. One solution to this problem is to use more accurate QM calculations for a small but interesting part of the structure (system 1), i.e., an approach similar to QM/MM calculations.^40,41^ This leads to a refinement target of the form
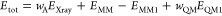
4where index 1 indicates calculations only of system 1. This represents the energy function of the quantum refinement. As crystallographic force fields are of a statistical nature, whereas the QM calculations are in energy units, another scaling factor, *w*_QM_, needs to be introduced to put the restraints on a similar level. We recently implemented^64^eq 4 in the *cctbx*(65) layer of the crystallographic refinement software *phenix.refine*,^66^ utilizing the free QM software ORCA 5.0.4^67,68^ to calculate *E*_QM1_.

In this study, we have used this new version of quantum refinement to study the protonation state of the homocitrate ligand in the crystal structure of Fe-nitrogenase (8BOQ).^13^ Coordinates, occupancies, *B*-factors, and structure factors were obtained from this structure. We used the minimal QM model in Figure 1a for the QM calculations with the TPSS-D4/def2-SV(P) method^30,31,57^ in the singlet state and BS state 2358 (obtained with the Flipspin approach in ORCA). Depending on the protonation state of the homocitrate ligand (see Figure 2), the net charge of the QM system was either −7 (0H), −6 (1Ha and 1Hc), or −5 (2H). The occupancy of S2B was set to 1.00, while the oxygen atom replacing S2B in the other conformation was discarded. Gln-176 was modeled with dual conformations as in the PDB file. Protonation of system 1 was done with *phenix.ready_set*. Restraint files for the nonstandard ligands (homocitrate, the P-cluster, and the FeFe cluster) were generated using *phenix.elbow*.^69^ Three macrocycles of combined coordinate and standard individual *B*-factor refinement in *phenix.refine* were performed, in which only Cys-257, His-423, homocitrate, and the FeFe cluster were allowed to move, after which the real-space *Z*-scores based on the difference maps (RSZD), the real-space *R* factors (RSR), and the real-space correlation coefficients (RSCCs) for homocitrate were calculated by the use of *EDSTATS*.^70^ We tested different values of the *w*_A_ weight factor in eq 4 and selected a value for which the structures were significantly affected by both the crystallographic and QM data, *w*_A_ = 1.0, cf. Table S9.

## Results and Discussion

We here present the first QM/MM study of Fe-nitrogenase, based on the recent crystal structure.^13^ We determine the most stable BS states for the resting state and the proper protonation states for the homocitrate ligand and for the catalytic His-180 residue. In addition, we have studied the protonation of the one-electron reduced E_1_ state.

### BS States of the E_0_ Resting State

We started by investigating which is the most stable BS state of the FeFe cluster in the resting E_0_ state. The cluster contains eight Fe ions, all of which have a high-spin configuration. However, the spins couple antiferromagnetically to a singlet state.^71^ Thus, four of the Fe ions have a surplus of α spin, and the other four have a surplus of β spin. Four Fe ions can be selected out of eight in 70 different ways , twice as many as for Mo- and V-nitrogenase. However, for the resting state, with an equal number of Fe(II) and Fe(III) ions, there is no distinction between the α and β electrons, and therefore, only 35 states are distinct (i.e., state 1234 is equivalent to the 5678 state, and so on; this was confirmed by explicit calculations, cf. Table S10). In the following, we present the results only for states with a surplus of β spin on Fe8. The relative energies of all these states were studied using the minimal 56-atom QM model (Figure 1a) with two DFT functionals: TPSSh and r^2^SCAN.

The relative energies are presented in Table 1. It can be seen that the results with the two functionals are quite consistent, with mean signed and mean absolute differences (MAD) for the relative energies of only 2 and 4 kJ/mol. Three states are lowest in energy and almost degenerate (within 3–4 kJ/mol): BS-2358, 3468, and 2478 (the numbers denote the Fe ions with β spin; atom numbering shown in Figure 1a). These correspond to the three BS7 in the Noodleman nomenclature for the MoFe cluster,^13^ with the extra Fe8 ion having a surplus of β spin. As can be seen from the schematic picture in Figure 3, these are the spin configurations that give the largest number of antiferromagnetically coupled pairs of nearby Fe ions (always for the Fe2–Fe6, Fe3–Fe7, and Fe4–Fe5 pairs, connecting the two Fe_4_S_4_ subclusters and two of the three pairs within each subcluster, Fe1–Fe2/3/4 and Fe8–Fe5/6/7). The fourth-best state is 16–20 kJ/mol less stable than the best state, BS-2378 with r^2^SCAN or BS-1258 with TPSSh. The ordering of the states follows approximately the Noodleman nomenclature^13^ with the order BS7 < BS10 ≈ BS8 < BS9 ≈ BS4 < BS6 ≈ BS5 < BS3 < BS1, with an ambiguity only for the BS2 state, which is similar to BS3 for TPSSh but similar to BS5 with r^2^SCAN. This order is rather different from what was observed for the E_0_ state of Mo-nitrogenase: BS7 < BS6 < BS2 < BS8 < BS4 ≈ BS10 < BS9 < BS5 < BS3 < BS1.^53^

**Table 1:** Relative Energy (Δ*E* in kJ/mol), Average (av), and Maximum (max) Metal–Metal and Meta–Ligand Distance Deviation from the Crystal Structure for the Various BS States of the Resting State^a^

BS	N	r^2^SCAN							TPSSh						
		Δ*E*	metal–metal			metal–ligand			Δ*E*	metal–metal			metal–ligand		
			av	max	bond	av	max	bond		av	max	bond	av	max	bond
1238	3	91.9	0.096	0.220	Fe5–Fe8	0.065	0.202	Fe8–S4B	96.7	0.081	0.177	Fe5–Fe8	0.061	0.131	Fe5–S4B
1248	3	81.1	0.103	0.179	Fe1–Fe4	0.067	0.199	Fe8–S4B	84.4	0.102	0.250	Fe5–Fe8	0.061	0.154	Fe8–S4B
1258	10	19.3	0.059	0.130	Fe6–Fe8	0.061	0.168	Fe8–S4B	20.4	0.065	0.136	Fe6–Fe8	0.059	0.148	Fe8–S4B
1268	9	49.7	0.104	0.216	Fe5–Fe7	0.065	0.149	Fe8–S1B	50.3	0.109	0.221	Fe5–Fe7	0.062	0.142	Fe5–S4B
1278	10	21.5	0.053	0.134	Fe5–Fe8	0.060	0.156	Fe8–S4B	22.7	0.060	0.161	Fe5–Fe8	0.057	0.139	Fe8–S4B
1348	3	87.4	0.097	0.206	Fe6–Fe8	0.061	0.176	Fe8–S1B	74.5	0.083	0.237	Fe5–Fe8	0.060	0.153	Fe5–S4B
1358	10	27.9	0.052	0.129	Fe6–Fe8	0.064	0.174	Fe8–S4B	27.0	0.057	0.131	Fe6–Fe8	0.061	0.155	Fe8–S4B
1368	10	26.5	0.057	0.146	Fe6–Fe8	0.064	0.145	Fe8–S1B	26.8	0.064	0.161	Fe5–Fe8	0.062	0.132	Fe8–S4B
1378	2	52.6	0.096	0.236	Fe2–Fe4	0.065	0.167	Fe8–S4B	49.2	0.098	0.237	Fe2–Fe4	0.063	0.150	Fe8–S4B
1458	3	43.0	0.110	0.222	Fe2–Fe3	0.063	0.179	Fe8–S4B	41.1	0.114	0.222	Fe2–Fe3	0.062	0.164	Fe8–S4B
1468	10	23.4	0.068	0.151	Fe5–Fe8	0.062	0.146	Fe8–S1B	22.8	0.079	0.175	Fe5–Fe8	0.060	0.128	Fe8–S4B
1478	10	28.6	0.054	0.138	Fe5–Fe8	0.059	0.158	Fe8–S4B	26.6	0.064	0.158	Fe5–Fe8	0.057	0.140	Fe8–S4B
1568	6	47.8	0.100	0.244	Fe6–Fe8	0.068	0.165	Fe8–S4B	57.3	0.102	0.241	Fe6–Fe8	0.066	0.148	Fe8–S4B
1578	6	53.9	0.102	0.200	Fe6–Fe8	0.068	0.197	Fe8–S4B	63.8	0.100	0.190	Fe6–Fe8	0.066	0.178	Fe8–S4B
1678	6	58,0	0.097	0.235	Fe6–Fe8	0.065	0.173	Fe4–C	66.7	0.098	0.235	Fe6–Fe8	0.062	0.156	Fe4–C
2348	2	59.4	0.099	0.201	Fe5–Fe8	0.059	0.184	Fe8–S4B	81.9	0.090	0.240	Fe5–Fe8	0.056	0.170	Fe8–S4B
2358	7	0.8	0.040	0.119	Fe5–Fe8	0.049	0.169	Fe8–S4B	0.0	0.042	0.122	Fe5–Fe8	0.047	0.152	Fe8–S4B
2368	8	29.3	0.072	0.167	Fe5–Fe8	0.062	0.155	Fe8–S1B	28.7	0.074	0.175	Fe5–Fe8	0.058	0.137	Fe8–S4B
2378	8	15.6	0.088	0.162	Fe2–Fe3	0.061	0.168	Fe8–S4B	20.8	0.089	0.165	Fe2–Fe3	0.057	0.152	Fe8–S4B
2458	8	25.8	0.070	0.142	Fe2–Fe4	0.063	0.182	Fe8–S4B	23.2	0.069	0.139	Fe2–Fe4	0.059	0.163	Fe8–S4B
2468	8	33.6	0.070	0.142	Fe6–Fe8	0.062	0.140	Fe8–S1B	32.0	0.070	0.147	Fe7–Fe8	0.058	0.134	Fe8–S4B
2478	7	3.9	0.033	0.088	Fe6–Fe8	0.051	0.152	Fe8–S4B	2.2	0.034	0.101	Fe5–Fe8	0.048	0.135	Fe8–S4B
2568	5	55.5	0.099	0.235	Fe1–Fe3	0.068	0.158	Fe8–S4B	61.2	0.096	0.211	Fe6–Fe8	0.065	0.144	Fe3–S4A
2578	4	45.0	0.079	0.197	Fe5–Fe8	0.058	0.173	Fe8–S4B	37.9	0.081	0.194	Fe5–Fe8	0.056	0.158	Fe8–S4B
2678	5	61.7	0.085	0.248	Fe5–Fe8	0.066	0.172	Fe8–S1B	57.2	0.090	0.248	Fe5–Fe8	0.063	0.163	Fe8–S1B
3458	8	28.9	0.055	0.138	Fe6–Fe8	0.061	0.168	Fe8–S4B	27.8	0.056	0.139	Fe6–Fe8	0.057	0.150	Fe8–S4B
3468	7	0.0	0.042	0.158	Fe6–Fe8	0.049	0.144	Fe8–S1B	2.6	0.042	0.139	Fe6–Fe8	0.045	0.114	Fe8–S4B
3478	8	28.9	0.062	0.129	Fe6–Fe8	0.058	0.151	Fe8–S4B	27.6	0.062	0.133	Fe6–Fe8	0.054	0.135	Fe8–S4B
3568	4	40.6	0.077	0.179	Fe6–Fe8	0.062	0.173	Fe8–S4B	50.5	0.080	0.171	Fe6–Fe8	0.060	0.163	Fe4–S4A
3578	5	57.1	0.099	0.227	Fe7–Fe8	0.071	0.194	Fe8–S4B	63.2	0.100	0.225	Fe7–Fe8	0.067	0.177	Fe8–S4B
3678	5	52.6	0.085	0.220	Fe5–Fe8	0.067	0.160	Fe8–S4B	58.4	0.082	0.237	Fe5–Fe8	0.063	0.144	Fe8–S1B
4568	5	62.4	0.103	0.259	Fe1–Fe3	0.068	0.177	Fe8–S4B	70.5	0.104	0.266	Fe1–Fe3	0.064	0.160	Fe8–S4B
4578	5	73.7	0.093	0.218	Fe6–Fe8	0.070	0.192	Fe8–S4B	79.8	0.089	0.225	Fe6–Fe8	0.065	0.173	Fe8–S4B
4678	4	61.6	0.077	0.198	Fe6–Fe8	0.057	0.159	Fe8–S1B	59.5	0.066	0.195	Fe6–Fe8	0.051	0.144	Fe8–S1B
5678	1	121.4	0.097	0.215	Fe1–Fe2	0.074	0.186	Fe8–S4B	122.6	0.091	0.206	Fe1–Fe2	0.072	0.170	Fe8–S4B

a Bond lists the bond that gives the maximum deviation. N is the type of the BS state in Noodleman’s nomenclature.

Fe spin populations are shown in Table S10. They are (in absolute terms) 3.4–3.8 *e* with TPSSh and 3.3–3.9 *e* for r^2^SCAN, with little variation among the eight Fe ions, besides that, Fe8 (which has six rather than four ligands) always has the highest spin population, by 0.2–0.3 *e*.

The various BS states give slightly different geometries of the FeFe cluster. Therefore, we can compare which of the BS states reproduces the crystal structure best in terms of the short Fe–Fe and Fe–ligand distances. The results in Table 1 show that the three BS7 states also reproduce the structure best, with mean absolute deviations of 0.03–0.04 and 0.05 Å for the Fe–Fe and Fe–ligand distances.

Based on these findings, we rather arbitrarily decided to make further studies of the E_0_ state with the BS-2358 state (BS-235), which has been argued to be the proper BS state for the E_0_ of Mo-nitrogenase.^72^

### Protonation State of Homocitrate and His-180

Next, we studied the protonation of the resting E_0_ state using QM/MM calculations. This was done with the larger 186-atom QM region, as shown in Figure S1. We investigated four different protonation states for HCA (shown in Figure 2) and three different protonation states for His-180. We also tested to protonate S2B.

In the crystal structure, all oxygen atoms of homocitrate are involved in hydrogen bonds with water molecules or with the surrounding residues. O1 receives hydrogen bonds from the NZ atom Lys-361 and NE2 of Gln-176 (in both of its conformations), whereas O2 forms two hydrogen bonds to water molecules (which form hydrogen bonds to other water molecules), and it is 2.99–3.17 Å from SG of Cys-52. O3 receives a hydrogen bond from the backbone N atom of Lys-406 and forms hydrogen bonds to two water molecules. O4 forms hydrogen bonds to three water molecules. O5 is an Fe ligand (the Fe–O distance is 2.08 or 2.36 Å in the two subunits) and forms a hydrogen bond to a water molecule, whereas O6 forms hydrogen bonds to two water molecules. Finally, the alcohol atom O7 is also a Fe ligand (2.09 or 2.17 Å distance), and it is 2.69–2.70 Å from the O1 atom. The NE2 atom of His-180 forms a hydrogen bond with the OE1 atom of Gln-176 in one of its two conformations (both have occupancies of 0.5). It is 3.33–3.45 Å from S2B which is also half-occupied and 3.54–3.62 Å from the alternative O atom. The ND1 atom forms a hydrogen bond with a water molecule, which forms hydrogen bonds to the OG of Ser-176 and the OH of Tyr-262 (which both can be either donors or acceptors).

In the MD equilibration of the protein for the QM/MM calculations, the O atom replacing S2B was deleted, together with the corresponding alternative conformation of Gln-176 and the storage position of S2B. After the equilibration, all water molecules donate hydrogen bonds to the carboxylate oxygen atoms of homocitrate (which were modeled unprotonated), giving H···O distances of 1.70–1.91 Å. The other hydrogen bonds also fall in the same range, except that between O1 and HE2 of Gln-175, which is 2.25 Å (all in the A subunit, used for the QM/MM calculations; 1.67–1.97 and 2.41 Å; and 2.06 Å to Lys in the D subunit). Adding a proton on the alcoholic O7 has no impact on the surroundings as it points toward O1 and does not interact with anything else in the surroundings. Likewise, a proton added to O2 does not interfere with the two water molecules forming hydrogen bonds to it. However, protonation of the ND1 of His-180 would require the imidazole group to rotate or a change in the hydrogen-bond network involving a water molecule, Ser-176 and Tyr-262.

**Figure 4:**
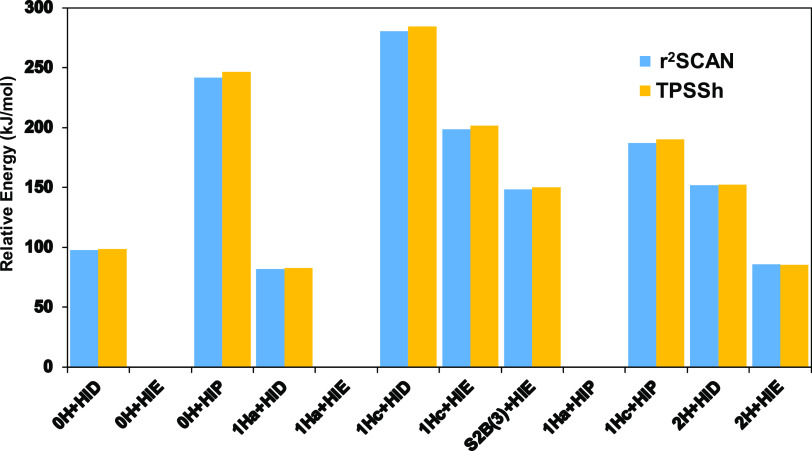
Relative energies (kJ/mol) of the various protonation states tested for Fe-nitrogenase in the E_0_ state.

The relative QM/MM energies of the various protonation states are presented in Figure 4. It can be seen that the two DFT methods give very similar results with a MAD of only 2 kJ/mol. Of course, energies are comparable only for structures with the same number of protons. Therefore, the data must be divided into three groups with one, two, or three protons that are moved around.

The first group involves only two structures, with no extra proton on homocitrate (0H) and with the proton on His-180 either on ND1 (called HID) or on NE2 (HIE). It can be seen that the latter state is most stable, ∼99 kJ/mol.

In the second group, involving six structures, an extra proton is put either on His-180 (called HIP) on S2B or on homocitrate, on either the O2 carboxylate atom (called 1Hc), or on the O7 alcohol atom (called 1Ha). The results show that the HIE state is always ∼82 kJ/mol more stable than the HID state. Moreover, protonation of the homocitrate alcohol atom is 202, 247, and 150 kJ/mol more favorable than protonating the carboxylate atom, His-180 or S2B. Thus, the 1Ha + HIE structure is by far the most stable structure in this group.

Finally, we also tested adding a third proton to the structures, which could either go to His-180 or to homocitrate (2H), giving four tested structures. The results show that it is more favorable to protonate His-180 than homocitrate. Moreover, HIE is still ∼67 kJ/mol more stable than HID and 1Ha is ∼190 kJ/mol more stable than 1Hc. Consequently, the 1Ha + HIP structure is the most stable for this protonation level.

The relative energies give no indication of which of the three groups is most favorable, i.e., how many protons the structure should contain. However, we can again compare the optimized structures with the crystal structure. This is done in Table 2, from which it can be seen that the 1Ha + HID and 1Ha + HIE structures reproduce the crystal structure best, with MADs of 0.04 and 0.05 Å for the Fe–Fe and Fe–ligand distances. The third-best structure is 1Ha + HIP, showing that the structure of the FeFe cluster is insensitive to the protonation of His-180, but that homocitrate is most likely singly protonated on the O7 alcohol atom (1Ha).

**Table 2:** Relative Energy (Δ*E* in kJ/mol), Average (av), and Maximum (max) Metal–Metal and Meta–Ligand Distance Deviations from the Crystal Structure for the QM/MM Structures of the Various Protonation States of the E_0_ Resting State^a^

	r^2^SCAN							TPSSh						
	Δ*E*	metal–metal			metal–ligand			Δ*E*	metal–metal			metal–ligand		
		av	max	bond	av	max	bond		av	max	bond	av	max	bond
0H + HID	98.0	0.065	0.25	Fe5–Fe8	0.061	0.30	Fe8–O1	98.7	0.058	0.20	Fe5–Fe8	0.060	0.29	Fe8–O1
0H + HIE	0.0	0.062	0.22	Fe5–Fe8	0.060	0.30	Fe8–O1	0.0	0.058	0.19	Fe7–Fe8	0.058	0.29	Fe8–O1
0H + HIP	241.6	0.069	0.26	Fe5–Fe8	0.059	0.31	Fe8–O1	246.6	0.062	0.22	Fe5–Fe8	0.061	0.31	Fe8–O1
1Ha + HID	81.7	0.039	0.08	Fe6–Fe8	0.045	0.12	Fe8–S4B	82.8	0.038	0.07	Fe5–Fe8	0.048	0.14	Fe8–S4B
1Ha + HIE	0.0	0.040	0.09	Fe6–Fe8	0.045	0.12	Fe8–S4B	0.0	0.039	0.09	Fe6–Fe8	0.047	0.13	Fe8–S4B
1Hc + HID	280.7	0.054	0.18	Fe7–Fe8	0.056	0.23	Fe8–O1	284.4	0.050	0.15	Fe7–Fe8	0.056	0.22	Fe8–O1
1Hc + HIE	198.8	0.056	0.18	Fe7–Fe8	0.055	0.23	Fe8–O1	201.8	0.051	0.15	Fe7–Fe8	0.056	0.22	Fe8–O1
S2B + HIE	148.2	0.068	0.27	Fe5–Fe8	0.061	0.32	Fe8–O1	150.1	0.064	0.23	Fe5–Fe8	0.062	0.32	Fe8–O1
1Ha + HIP	0.0	0.044	0.13	Fe6–Fe8	0.046	0.12	Fe8–S4B	0.0	0.043	0.12	Fe6–Fe8	0.048	0.14	Fe8–S1B
1Hc + HIP	187.3	0.061	0.18	Fe7–Fe8	0.056	0.24	Fe8–O1	190.0	0.055	0.15	Fe7–Fe8	0.057	0.24	Fe8–O1
2H + HID	151.8	0.040	0.12	Fe6–Fe8	0.049	0.18	Fe8–S3B	152.5	0.039	0.123	Fe6–Fe8	0.052	0.15	Fe7–S3B
2H + HIE	86.1	0.041	0.13	Fe6–Fe8	0.051	0.14	Fe8–S3B	85.6	0.040	0.14	Fe6–Fe8	0.053	0.15	Fe7–S3B

a All calculations were performed in the 2358 BS state. Bond shows the bond that gives the maximum deviation.

The Fe–O7 distance is 2.13–2.15 Å when it is protonated in the QM/MM structures (2.32–2.35 Å when O2 is also protonated), but 1.90–1.93 (0H) or 1.98–1.99 Å (H1c) when it is deprotonated. The Fe–O5 distance is 2.14–2.22 Å in the various structures. As mentioned above, there is a large variation in the distances in the two subunits of the crystal structure (2.08–2.36 Å), but it is never close to the distance of the deprotonated alcoholate ligand.

To further strengthen this important conclusion, we also performed quantum refinement of the E_0_ state of Fe-nitrogenase with the four protonation states of homocitrate. The results of these refinements are shown in Table 3. It can be seen that all three crystallographic quality measures (RSZD, RSR, and RSCC) for homocitrate are best for the 1Ha protonation state, in agreement with the QM/MM data. This is also confirmed by the electron-density difference maps around the homocitrate ligand for the four quantum refinements, as shown in Figure S2. The difference in the Fe–O bond lengths to the two O atoms of homocitrate between the quantum-refined structure and a structure optimized without any restraints to the crystal structures (i.e., a QM/MM structure with the MM force field used by Phenix) is also the smallest for the 1Ha protonation state. In particular, it can be seen that the Fe–O7 distance to the alcohol atom in the quantum-refined structures is always longer than the expected bond length if it is deprotonated, 1.99–2.06 Å. However, the strain energy (Δ*E*_str_), i.e., the difference in the QM energy of the QM region between the quantum-refined structure and the structure optimized without any crystallographic restraints, is lowest for the 2H protonation state, but Δ*E*_str_ is comparable only for structures with the same net charge, i.e., in this case only for 1Ha and 1Hc, for which 1Ha gives the better results. In conclusion, the quantum-refinement calculations also quite conclusively point out 1Ha as the protonation state observed in the crystal structure of Fe-nitrogenase in the E_0_ state.

**Table 3:** Quality Measures and Fe–O Distances (*d* in Å) for the Four Quantum-Refined Structures with Varying Protonation States of Homocitrate (cf. Figure 2)^a^

	RSZD	RSR	RSCC	Δ*E*_str_ (kJ/mol)	ΣΔ*d* (Å)	*d*(Fe–O)_QR_		*d*(Fe–O)_QM_	
						O5	O7	O5	O7
2H	1.1	0.046	0.962	35	0.08	2.22	2.21	2.23	2.28
1Ha	**0.5**	**0.044**	**0.967**	41	**0.07**	2.17	2.20	2.13	2.23
1Hc	1.7	0.050	0.955	54	0.08	2.23	2.13	2.22	2.06
0H	2.8	0.055	0.944	38	0.10	2.22	2.06	2.19	1.99

a The quality measures are the real-space *Z*-scores based on the difference maps (RSZD), the real-space *R* factors (RSR), the real-space correlation coefficients (RSCC) for homocitrate, the strain energy of the QM region (Δ*E*_str_), and the sum of the difference in the Fe–O bond lengths (ΣΔ*d*) to the homocitrate O5 (carboxylate) and O7 (alcohol) atoms in the quantum-refined structure [*d*(Fe–O)_QR_] and in a structure optimized by QM/MM without any crystallographic information [*d*(Fe–O)_QM_]. The best results of each quality measure are marked in bold face.

Finally, we also performed MD simulations of Fe-nitrogenase with different protonation states of homocitrate and His-180. The hydrogen-bond pattern in these simulations is shown in Tables S11–S16. In the preferred protonation state (1Ha for homocitrate and His-180 protonated on NE2), the proton on O7 of homocitrate forms an internal hydrogen bond to O1 (like in the QM/MM and quantum-refined structures). O1 also receives hydrogen bonds from the HZ atoms Lys-361, from one of the HE2 atoms of Gln-176 and from water molecules, with rather large variation between the two subunits of the protein. O2 forms hydrogen bonds to 2–3 water molecules. O3 and O4 receive a hydrogen bond from the backbone N atom of Lys-406 and form hydrogen bonds to 2–3 water molecules (they show similar patterns and thus rotate during the MD simulation). O5 forms a hydrogen bond to a water molecule, whereas O6 forms hydrogen bonds to two water molecules. The HE2 atom of His-180 forms a hydrogen bond to S2B in most snapshots (∼2.4 Å average distance). The ND1 atom forms a hydrogen bond with a water molecule in 54–71 of the snapshots and occasionally with the backbone H atom of the same residue.

When instead ND1 of His-180 is protonated, the HD1 proton donates hydrogen bonds to either the backbone O atom or to a water molecule (observed in 46–51 and 30–40% of the MD snapshots, respectively). The NE2 atom sometimes receives a hydrogen bond from a water molecule. If both the ND1 and NE2 atoms are protonated, the same hydrogen bonds are observed, but with higher occurrences (72–87 and 34–64%).

If instead, O2 is protonated in homocitrate, the proton forms occasional hydrogen bonds to a water molecule (23–35% occurrences). The unprotonated O7 receives hydrogen bonds from the HZ atoms of Lys-361 and occasionally from water.

The assignment of the 1Ha protonation state for homocitrate (i.e., singly protonated on the alcohol oxygen) agrees with a previous quantum-refinement study of Mo-nitrogenase,^54^ as well as a comparison of the QM/MM and crystal structures of this enzyme.^73^ Comparisons of vibrational and CD spectra, as well as crystallographic structures of model compounds and the extracted cofactor of Mo- and V-nitrogenase, have given the same results.^74−76^

### Protonation of the E_1_ State

Once we have settled the proper protonation states of homocitrate and His-180 and the BS state for E_0_, we can turn to the main subject of the present investigation, viz., the protonation of the E_1_ state.

**Figure 5:**
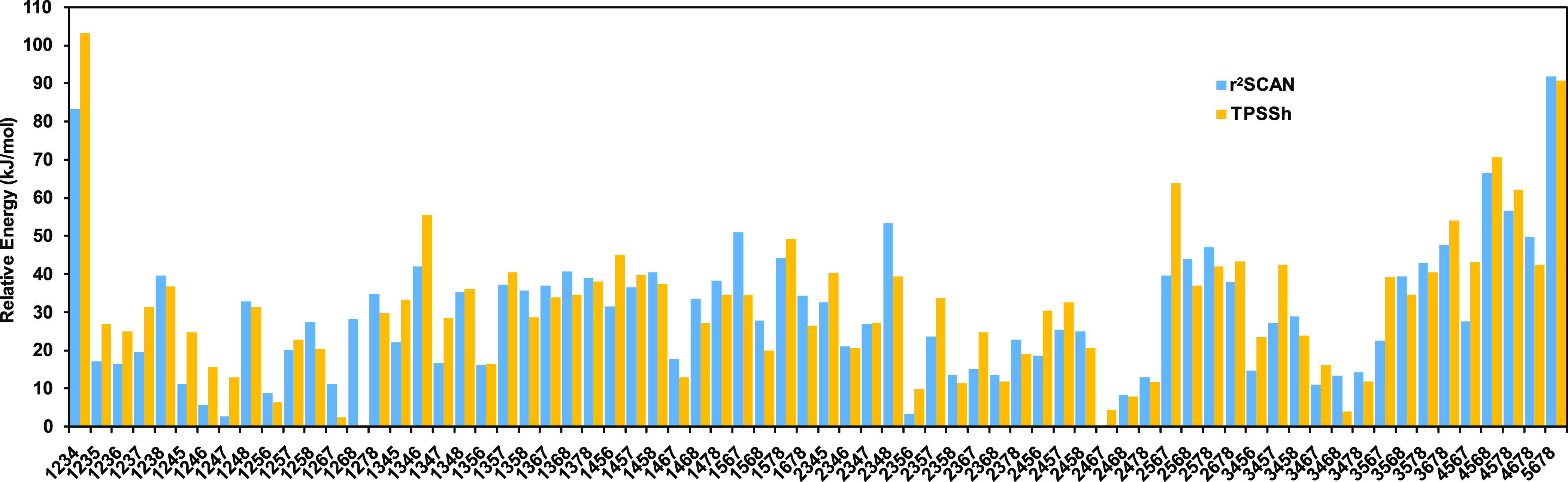
Relative energies (kJ/mol) of the various BS states of Fe-nitrogenase in the E_1_ state, protonated on S2B(3), using the minimal QM region.

First, we tested again what BS state is most favorable (with the extra proton on S2B). The results are shown in Figure 5 and Table S17. For E_1_, the 70 BS states are distinct, with mean absolute deviations of 16–17 kJ/mol between states for which the Fe ions with majority α and β spin have been swapped (e.g., BS-1234 and BS-5678; for E_0_, the difference was in general less than 1 kJ/mol). It can be seen that there is a larger difference between the r^2^SCAN and TPSSh functionals than that for E_0_, with a MAD of 7 kJ/mol (4 kJ/mol for E_0_). With r^2^SCAN, seven BS states give energies that are degenerate within 9 kJ/mol, viz., BS-2467, 2356, 1247, 1246, 2468, 1256, and 1268 (in this order). They belong to Noodleman’s BS4, 5, 8, 9, and 10 types but do not include the BS7 states that were lowest for E_0_ (they are 13–20 kJ/mol less stable than the best BS-2467 state). With TPSSh, the most stable BS state is 1268 (which is 4 kJ/mol more stable than the 2467 state), and the same states are among the most stable ones (although the ordering is different), including also BS-1267 and 3468 (the latter one of the BS7-type states).

Next, we calculated the relative energies of 50 different protonation states for the E_1_ state of the FeFe cluster with both functionals (using BS-2468). This involved protonation of Cys-257, His-180, homocitrate, the central carbide (with the proton on the three different faces of the cluster), sulfide ions (at least two different conformations of each), Fe ions, or bridging two Fe ions (typically two different conformations).^23^

**Figure 6:**
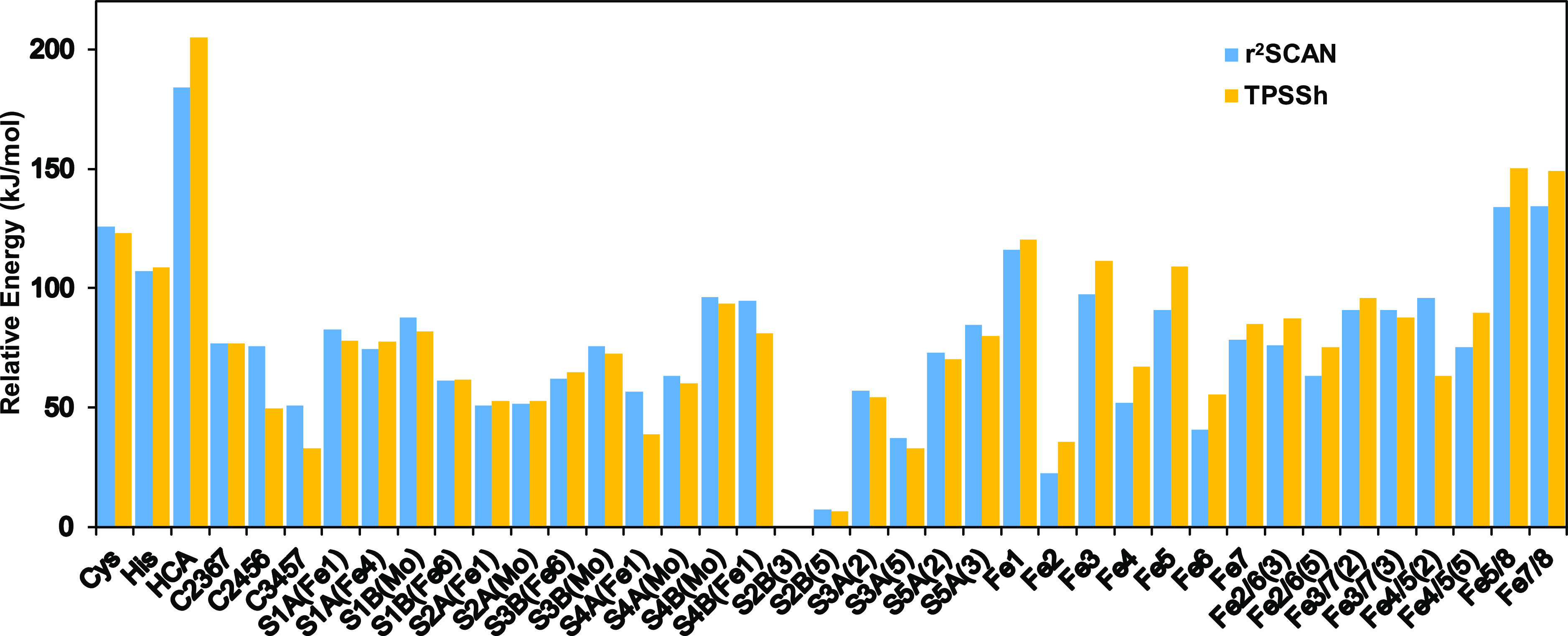
Relative energies (kJ/mol) of the various protonation states of Fe-nitrogenase in the E_1_ state using the large QM region and the BS-2468 state.

**Figure 7:**
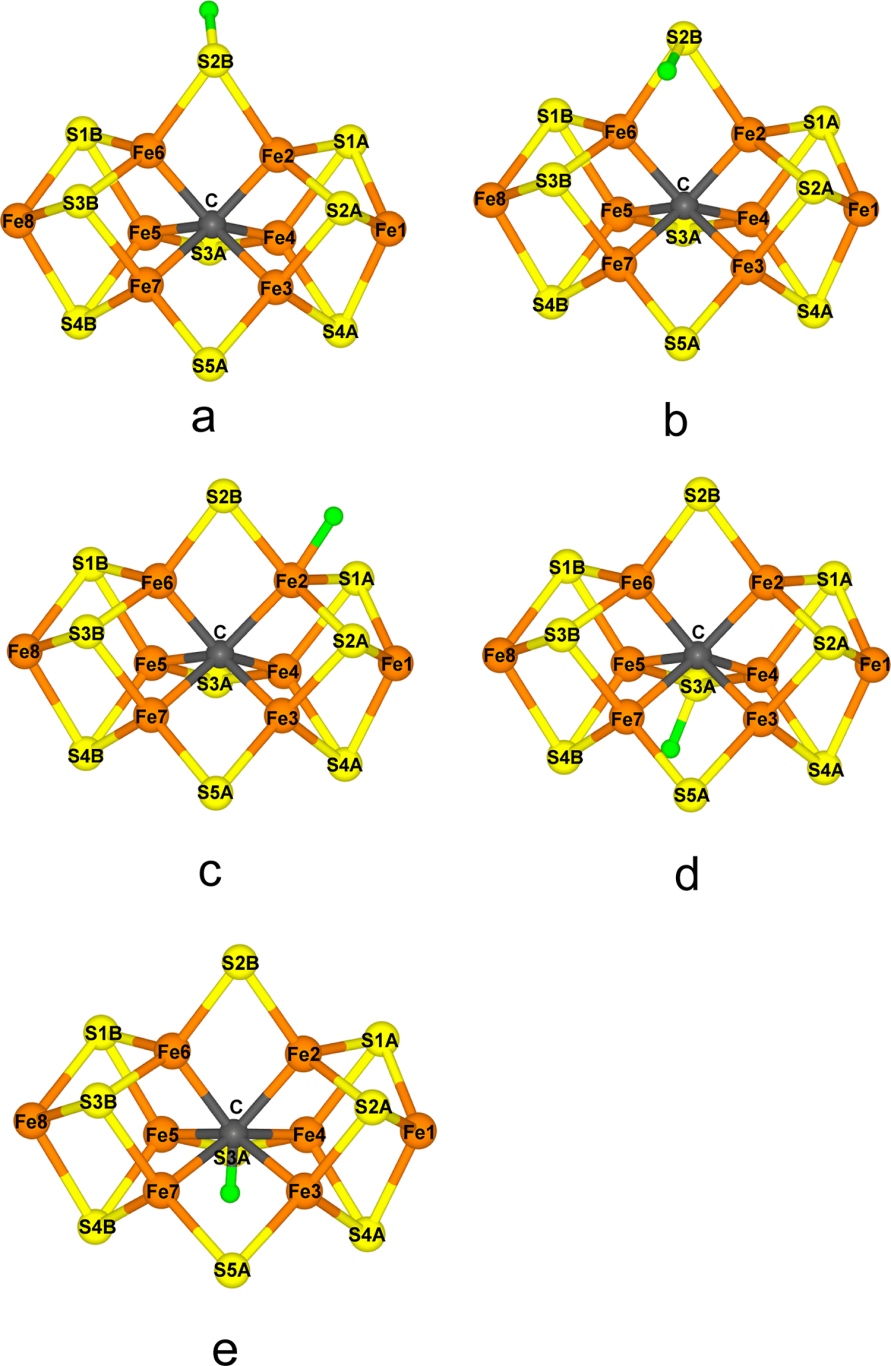
Best QM/MM structures of the E_1_ state of Fe-nitrogenase, protonated on (a) S2B(3), (b) S2B(5), (c) Fe2, (d) S3A(5), and (e) C3457, all optimized with the r^2^SCAN functional (with the 1Ha state of homocitrate and the HIE state of His-180).

The results are collected in Figure 6 and Table S18. With both functionals, protonation of S2B is most favorable. We tested two directions of this proton, and it turns out that it is 6–7 kJ/mol more favorable if the proton points toward S3A (S2B(3)), rather than toward S5A (S2B(5)), cf. Figure 7a,b. In this conformation, the added proton is rather close to Phe-361 (2.21 Å between the proton and CZ; however, the SB2–CZ distance has only increased by 0.1 Å compared to the other structures). Besides this, there is some discrepancy between the two functionals. In general, TPSSh shows a tendency to favor protonation of the central carbide and disfavor protonation of one or two Fe ions. Consequently, with r^2^SCAN, protonation of Fe2 (Figure 7c) is third best, 22 kJ/mol less stable than S2B(3), and protonation of Fe6 and Fe4 is among the nine best structures, whereas these structures rank 5, 11, and 15 with TPSSh. Instead, protonation of S3A(5) (also a μ_2_ belt sulfide; Figure 7d) or the central hydride on the Fe3/4/5/7 face (called C3457; Figure 7e) is the third and fourth best structure, both 33 kJ/mol less stable than S2B(3). These two structures rank four and seven with r^2^SCAN. Next come several structures with μ_3_ cubane sulfide ions protonated, especially S2A and S4A (both in the Fe1–4 subcluster). The best structure with a bridging hydride ion is Fe2/6(5) with both functionals, 63 or 75 kJ/mol less stable than the best structure. The least favorable structures have the proton on His-180, Cys-257, homocitrate, or bridging two Fe ions within the same subcluster (most structures of the latter type were not found).

We also repeated the complete BS investigation using the large 188-atom model for both the S2B(3) and Fe2-protonated structures and the r^2^SCAN, TPSSh, B3LYP, and TPSS functionals. The results are shown in Tables S19–S22. The MAD in the relative BS energies obtained with the large and minimal models is 5–7 kJ/mol for the r^2^SCAN and TPSSh functionals. With all four functionals, S2B(3) is more stable than the Fe2 structure by 26, 32, 117, and 14 kJ/mol, respectively, reflecting that iron-bound hydride ions are increasingly disfavored as the amount of Hartree–Fock exchange increases in the hybrid functionals. The most stable BS state also varies among the four functionals, BS-2467, 1256, 2368, and 3468 for the S2B(3) structure, and BS-2458 (B3LYP) or 2358 (the other functionals) for the structure with a hydride ion on Fe2. Many of the Fe2 structures reorganized into other structures during the geometry optimization, especially with B3LYP. There are many BS states with similar energies, especially with the pure functionals and the S2B(3) state (e.g., 9, 9, 2, and 5 states within 10 kJ/mol of the best one for S2B(3) and 6, 2, 2, and 7 for Fe2 with the four functionals, respectively).

Finally, we calculated the relative energies of five of the best protonation states also with the larger def2-TZVP basis set (full geometry optimizations) and with the surrounding relaxed, with both the r^2^SCAN and TPSSh functionals. The results are collected in Table 4. It can be seen that increasing the basis set has a minor influence on the relative energies, by up to 3 kJ/mol for r^2^SCAN and up to 11 kJ/mol with TPSSh. However, this leads to slight changes in the ranking of the five states with TPSSh (S2B(5) is now 1 kJ/mol more stable than the S3B(3) structure and the Fe2, S3A(5) and C3457 structures become essentially degenerate). If the surrounding protein and solvent are allowed to relax, similar restricted changes in the relative energies are observed (but this time larger for r^2^SCAN than for TPSSh, up to 12 and 5 kJ/mol, respectively). With both functionals, the S2B(3) state is stabilized compared to the other states, and with r^2^SCAN, also the Fe2 and C3457 states. However, neither the basis set nor the relaxation of the surroundings change the conclusion that protonation of S2B is 26–37 kJ/mol more favorable than a hydride ion on Fe2.

**Table 4:** Relative Energies (kJ/mol) of the Five Structures in Figure 7 Calculated with Either r^2^SCAN or TPSSh and the Larger def2-TZVP Basis Set (TZ) or with the Original def2-SV(P) Basis Set (SV) and with the Surrounding Protein and Water Molecules Relaxed (Relax)

DFT	protonation	BS	SV	TZ	SV-relax
r^2^SCAN	S2B(3)	2467	0	0	0
	S2B(5)	2467	9	7	20
	Fe2	2358	26	26	29
	S3A(5)	2467	43	41	54
	C3457	2468	61	64	62
TPSSh	S2B(3)	1256	0	0	0
	S2B(5)	1256	6	–1	12
	Fe2	2358	32	30	37
	S3A(5)	2468	42	31	46
	C3457	1256	35	32	36

With Mo-nitrogenase,^23^ protonation of S2B(3) was found to be most stable, 7 (TPSS) or 35 (B3LYP) kJ/mol more stable than S2B(5). However, the most stable structure with a hydride ion was on Fe4, which was 27 or 96 kJ/mol (TPSS and B3LYP), less stable than that protonated on S2B(3). The Fe2 structure was 51 or 131 kJ/mol less stable than S2B(3). Moreover, the best BS state for S2B(3) was BS7-346, followed by the other two BS7 states, 24–33 kJ/mol higher in energy. Thus, there are significant differences between Fe and Mo-nitrogenases, especially regarding the preferred BS states.

## Conclusions

In this study, we have set up the first QM/MM calculations of Fe-nitrogenase. This involves determining the proper protonation states of all residues and MD relaxation of the added protons and the surrounding water molecules.

Then, we have examined all 70 BS states of the cluster in the resting E_0_ state, showing that the relative stabilities of the states are rather similar to those obtained for the FeMo cluster, although the order of the various BS states has some conspicuous differences. However, the most stable BS states are still of Noodleman’s BS7 type, together with a surplus of β spin on the eighth Fe ion, viz., BS-2358, 2478, and 3468, which are degenerate within 4 kJ/mol. These are the states that give the largest number of antiferromagnetically coupled close Fe–Fe pairs (Figure 3), and they also reproduce Fe–Fe and Fe–ligand distances best compared to the crystal structure. For the E_0_ state, there is no difference between α and β spins so there are only 35 distinct BS states.

Next, we investigated the protonation states of homocitrate and His-180. The relative QM/MM energies show that His-180 strongly prefers protonation on NE2 rather than on ND1. Moreover, homocitrate seems to be most stable when it is singly protonated on the alcohol atom, O7 atom. This finding is also supported by the quantum refinement of the crystal structure. In both cases, the preferred protonation states are the same as those found for Mo-nitrogenase.

Finally, we turned to the E_1_ state. For this state, the 70 BS states are distinct, and many BS states are close in energy. There are significant differences in the preferred BS states in the E_1_ state compared to those observed for Mo-nitrogenase. We optimized the structures of 50 different protonation states. The relative energies depend somewhat on what DFT method is used, but with all four functionals tested, protonation of the μ_2_ belt sulfide ion S2B is more favorable than the formation of a Fe-bound hydride ion. A hydride bound terminally to Fe2 in the exo position (trans to the carbide ion) is the best hydride-bound structure, but it is 14, 26, 32, and 117 kJ/mol less stable than the structure protonated on S2B with the TPSS, r^2^SCAN, TPSSh, and B3LYP functionals, respectively. This does not change if a larger basis set is used or if the surroundings are relaxed during the geometry optimization. Thus, our results indicate that the E_1_ state does not contain any Fe-bound hydride ion, in agreement with what has been found for Mo-nitrogenase,^22−24^ but contrary to a recent EPR results suggesting that the E_1_ state of Fe-nitrogenase involves a hydride ion.^21^ However, it should be noted that the experimental evidence is only indirect: it is observed that the EPR signal of the E_1_ state at 12 K is partly converted to another signal if illuminated with 450 nm light, leading to a ∼70/30% equilibrium at long times. The conversion has a kinetic isotope effect of 2.0–2.8. If the temperature is increased to 145 K, then the structure relaxes back to the original state. This is interpreted as a conversion between two hydride-bound states, which the authors suggest to be Fe2/6 and Fe3/7 structures. Clearly, these two structures are high in energy in our calculations, 63–96 kJ/mol less stable than the S2B(3) structure. Undoubtedly, more investigations are needed to settle the nature of the E_1_ state in Fe-nitrogenase.

## Supporting Information Available

Large QM system with residue labels, difference maps for the four quantum-refined structures of homocitrate, RESP charges used for the FeFe cluster, P-cluster and the Mg site, relative energies and spin densities for the various BS states for the E_0_ and E_1_ state in the S2B(3) and Fe2 protonation states, and various protonation states of E_1_ in the BS-2468 state (PDF)
Coordinates of the most stable states (ZIP)
