# Supplementary material for: Protonation of Homocitrate and the E1 State of Fe-Nitrogenase Studied by QM/MM Calculations
Source: Inorg Chem. 2023 Nov 21;62(48):19433–45. doi: 10.1021/acs.inorgchem.3c02329 (PMC10698722; doi:10.1021/acs.inorgchem.3c02329)
Supplement: Supplementary file 1 — ic3c02329_si_001.pdf [file ic3c02329_si_001.pdf]

Supporting Information

Protonation of Homocitrate and the E_1 State of Fe-Nitrogenase Studied by QM/MM Calculations

Hao Jiang, Kristoffer Lundgren and Ulf Ryde

Department of Computational Chemistry, Lund University, Chemical Centre,

P. O. Box 124, SE-221 00 Lund, Sweden

Correspondence to Ulf Ryde, E-mail: Ulf.Ryde@compchem.lu.se,

Tel: +46 – 46 2224502, Fax: +46 – 46 2228648

2023-09-14

Figure S1. The large QM system for the FeFe cluster in the E_0 state, illustrating also names of the nearby residues.

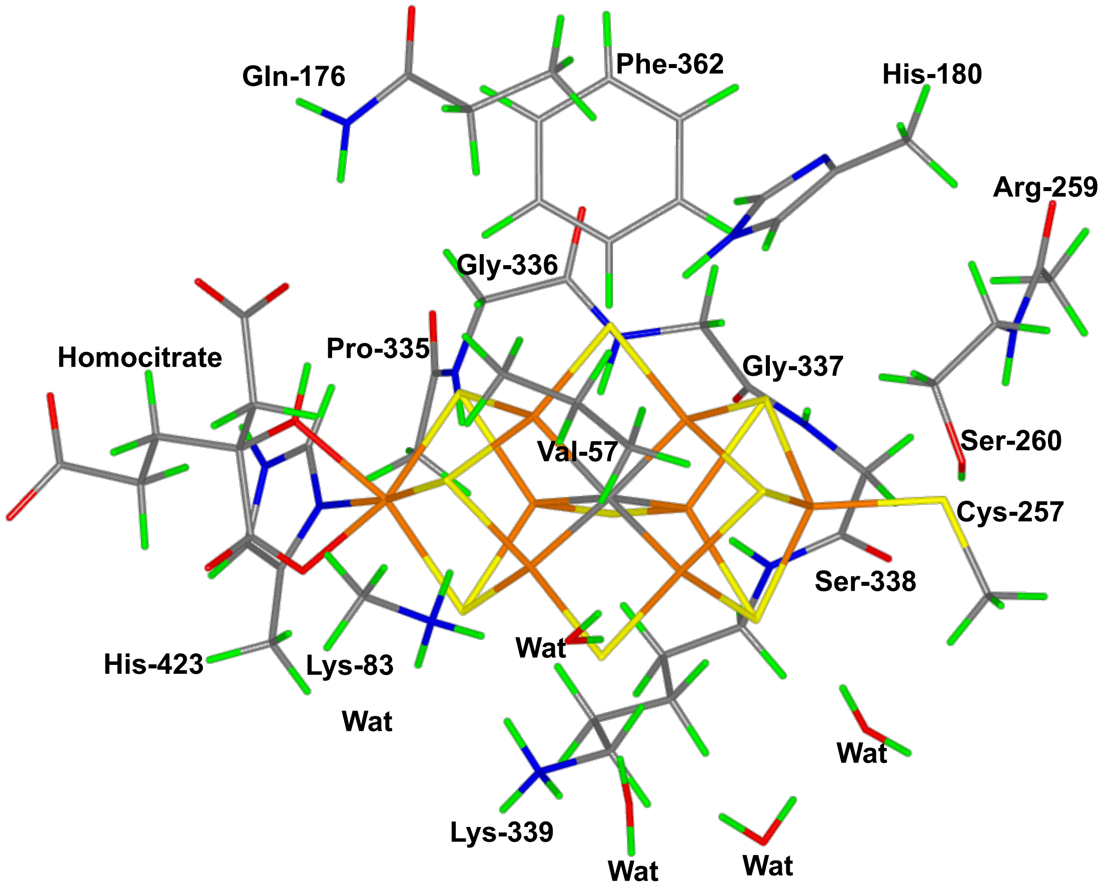

Figure S2. $mF_o - DF_c$ electron-density difference maps around the homocitrate ligand (+3σ in green and –3σ in red) from the quantum refinement in Fe-nitrogenase using the four different protonation states of homocitrate shown in Figure 2 in the main article: a) 2H, b) 1Ha, c) 1Hc and d) 0H.

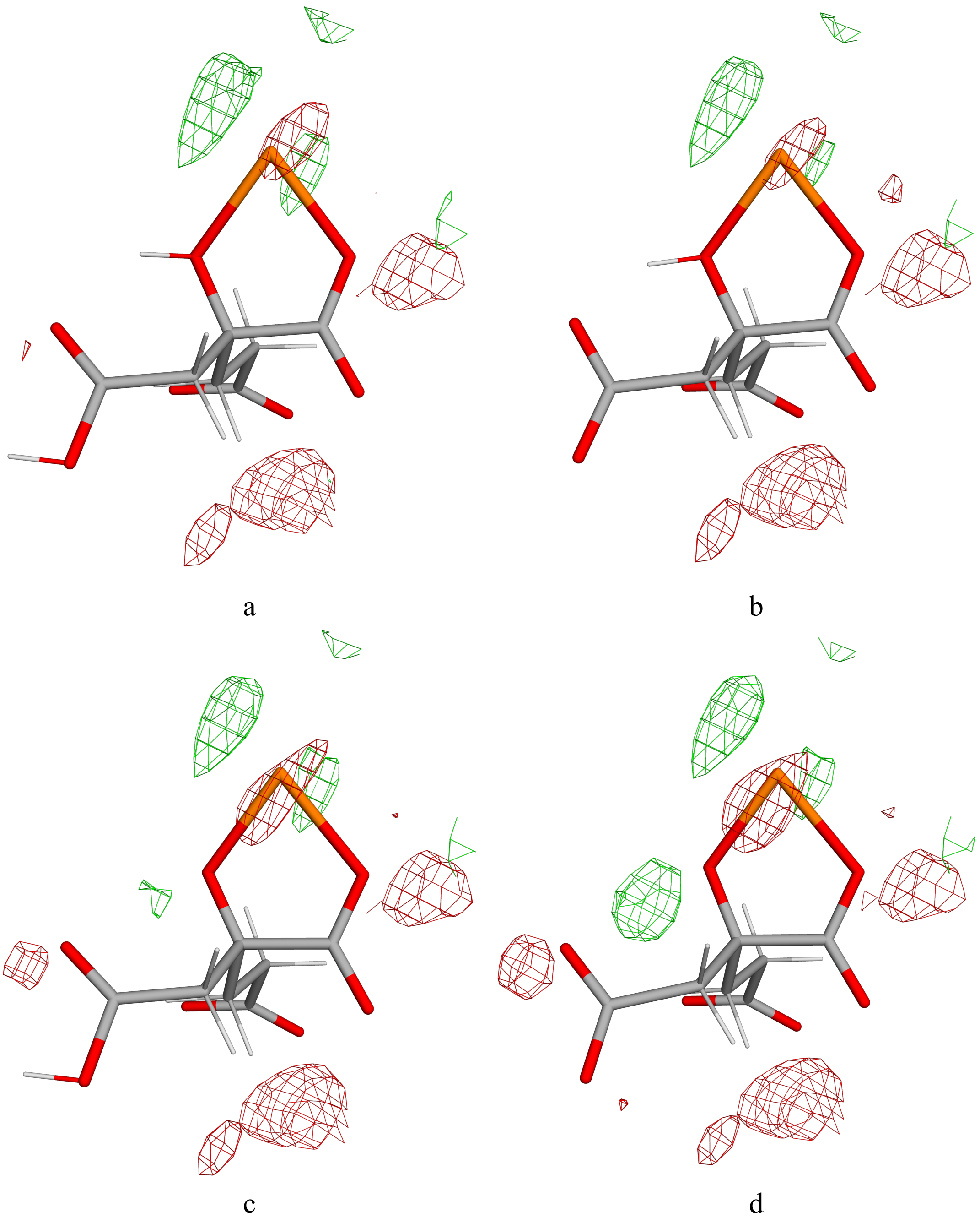

Table S1. RESP charges employed for the atoms in the FeFe cluster, homocitrate in the 0H state (net charge -7).

Cys-257		His-423		HCA		Cluster	
N	-0.415700	N	-0.415700	O3	-0.794095	FE1	0.592829
H	0.271900	H	0.271900	C6	0.620126	FE2	0.065045
CA	0.035925	CA	0.074122	O4	-0.789568	FE3	0.199966
HA	0.050800	HA	0.136000	C5	-0.072915	FE4	0.214318
CB	-0.103760	CB	-0.262992	H51	-0.034175	FE5	0.230536
HB3	0.014924	HB2	0.053121	H52	-0.034175	FE6	0.408322
HB2	0.014924	HB3	0.053121	C4	0.490484	FE7	0.299480
SG	-0.757224	CG	0.145586	H41	-0.220399	FE8	1.226649
C	0.597300	ND1	-0.009471	H42	-0.220399	C	0.337547
O	-0.567900	CE1	-0.091555	C3	0.445061	S1A	-0.646398
		HE1	0.176019	O7	-0.840446	S2A	-0.595314
		NE2	-0.261206	C7	0.729652	S3A	-0.797379
		HE2	0.302911	O5	-0.787862	S4A	-0.666642
		CD2	-0.377091	O6	-0.734372	S5A	-0.756337
		HD2	0.143951	C2	-0.067327	S1B	-0.759240
		C	0.597300	H21	-0.059632	S2B	-0.761633
		O	-0.567900	H22	-0.059632	S3B	-0.638542
				C1	0.647439	S4B	-0.712007
				O1	-0.756457		
				O2	-0.811812		

Table S2. RESP charges employed for the atoms in the FeFe cluster, homocitrate in the 1Ha state (net charge -6). These charges were used also for the equilibration of the protein for the QM/MM calculations.

Cys-257		His-423		HCA		Cluster	
N	-0.415700	N	-0.415700	O3	-0.787494	FE1	0.607545
H	0.271900	H	0.271900	C6	0.628230	FE2	0.191071
CA	0.108541	CA	0.085500	O4	-0.754559	FE3	0.276994
HA	0.050800	HA	0.136000	C5	-0.042842	FE4	0.292438
CB	-0.152991	CB	-0.439989	H51	-0.032654	FE5	0.317388
HB3	0.036623	HB2	0.115443	H52	-0.032654	FE6	0.414499
HB2	0.036623	HB3	0.115443	C4	0.237379	FE7	0.400473
SG	-0.703738	CG	0.228060	H41	-0.134487	FE8	0.988479
C	0.597300	ND1	0.071277	H42	-0.134487	C	0.045354
O	-0.567900	CE1	-0.140342	C3	0.470464	S1A	-0.643781
		HE1	0.172178	O7	-0.783132	S2A	-0.630142
		NE2	-0.180655	HO7	0.439459	S3A	-0.731956
		HE2	0.283641	C7	0.656481	S4A	-0.652751
		CD2	-0.383941	O5	-0.683254	S5A	-0.720346
		HD2	0.146193	O6	-0.704648	S1B	-0.754784
		C	0.597300	C2	-0.275261	S2B	-0.697771
		O	-0.567900	H21	0.005144	S3B	-0.689047
				H22	0.005144	S4B	-0.701339
				C1	0.734562		
				O1	-0.727878		
				O2	-0.751726		

Table S3. RESP charges employed for the atoms in the FeFe cluster, homocitrate in the 1Hc state (net charge -6).

Cys-257		His-423		HCA		Cluster	
N	-0.415700	N	-0.415700	O3	-0.796873	FE1	0.594135
H	0.271900	H	0.271900	C6	0.676224	FE2	0.202628
CA	0.055812	CA	0.093096	O4	-0.773342	FE3	0.274386
HA	0.050800	HA	0.136000	C5	-0.147243	FE4	0.288143
CB	-0.143407	CB	-0.294332	H51	-0.020814	FE5	0.302225
HB3	0.034812	HB2	0.072096	H52	-0.020814	FE6	0.413194
HB2	0.034812	HB3	0.072096	C4	0.192198	FE7	0.389751
SG	-0.708264	CG	0.166250	H41	-0.129119	FE8	0.990531
C	0.597300	ND1	0.078838	H42	-0.129119	C	0.06387
O	-0.567900	CE1	-0.121034	C3	0.895741	S1A	-0.650872
		HE1	0.168736	O7	-0.909539	S2A	-0.640043
		NE2	-0.206095	C7	0.578594	S3A	-0.737076
		HE2	0.291909	O5	-0.666571	S4A	-0.647799
		CD2	-0.386785	O6	-0.723435	S5A	-0.72211
		HD2	0.153571	C2	-0.401983	S1B	-0.75404
		C	0.597300	H21	0.049771	S2B	-0.720878
		O	-0.567900	H22	0.049771	S3B	-0.692977
				C1	0.546196	S4B	-0.689266
				O1	-0.587271		
				O2	-0.614769		
				HO2	0.348485		

Table S4. RESP charges employed for the atoms in the FeFe cluster, homocitrate in the 2H state (net charge -5).

Cys-257		His-423		HCA		Cluster	
N	-0.415700	N	-0.415700	O3	-0.766945	FE1	0.575176
H	0.271900	H	0.271900	C6	0.628822	FE2	0.210439
CA	0.069615	CA	0.136653	O4	-0.742742	FE3	0.271570
HA	0.050800	HA	0.136000	C5	-0.025176	FE4	0.289519
CB	-0.168295	CB	-0.407792	H51	-0.013553	FE5	0.263285
HB3	0.048614	HB2	0.115652	H52	-0.013553	FE6	0.326693
HB2	0.048614	HB3	0.115652	C4	0.117838	FE7	0.337905
SG	-0.662977	CG	0.169009	H41	-0.093365	FE8	0.926989
C	0.597300	ND1	-0.026545	H42	-0.093365	C	0.089472
O	-0.567900	CE1	-0.086657	C3	0.254326	S1A	-0.605764
		HE1	0.143241	O7	-0.458123	S2A	-0.595765
		NE2	-0.209681	HO7	0.291585	S3A	-0.686324
		HE2	0.306194	C7	0.741177	S4A	-0.605796
		CD2	-0.362420	O5	-0.695232	S5A	-0.666367
		HD2	0.166224	O6	-0.696398	S1B	-0.719879
		C	0.597300	C2	-0.271691	S2B	-0.667565
		O	-0.567900	H21	0.043321	S3B	-0.662930
				H22	0.043321	S4B	-0.640605
				C1	0.679486		
				O1	-0.479416		
				O2	-0.643981		
				HO2	0.400511		

Table S5. RESP charges employed for the atoms in the FeFe cluster, homocitrate in 1Ha state with an additional hydride ion on Fe2 (net charge -5).

Cys-257		His-423		HCA		Cluster	
N	-0.415700	N	-0.415700	O3	-0.744839	FE1	0.539080
H	0.271900	H	0.271900	C6	0.561140	FE2	0.102875
CA	0.073739	CA	0.130546	O4	-0.730974	FEH	-0.046644
HA	0.050800	HA	0.136000	C5	0.042946	FE3	0.254545
CB	-0.168012	CB	-0.405411	H51	-0.005502	FE4	0.305619
HB3	0.052740	HB2	0.109547	H52	-0.005502	FE5	0.266745
HB2	0.052740	HB3	0.109547	C4	-0.000636	FE6	0.360097
SG	-0.631896	CG	0.168573	H41	-0.059860	FE7	0.336921
C	0.597300	ND1	-0.027485	H42	-0.059860	FE8	1.098638
O	-0.567900	CE1	-0.107530	C3	0.396886	C	-0.029404
		HE1	0.161709	O7	-0.686190	S1A	-0.504544
		NE2	-0.219468	HO7	0.382539	S2A	-0.504466
		HE2	0.324276	C7	0.697152	S3A	-0.642878
		CD2	-0.374849	O5	-0.719720	S4A	-0.558653
		HD2	0.186129	O6	-0.684519	S5A	-0.606263
		C	0.597300	C2	-0.243826	S1B	-0.619952
		O	-0.567900	H21	0.005783	S2B	-0.453193
				H22	0.005783	S3B	-0.529808
				C1	0.732089	S4B	-0.602770
				O1	-0.710092		
				O2	-0.731638		

Table S6. RESP charges employed for the atoms in the FeFe cluster, homocitrate in 1Ha state with an additional proton on S2B (3) (net charge -5).

Cys-257		His-423		HCA		Cluster	
N	-0.415700	N	-0.415700	O3	-0.745299	FE1	0.527825
H	0.271900	H	0.271900	C6	0.566191	FE2	0.185536
CA	0.083630	CA	0.113143	O4	-0.731037	FE3	0.249982
HA	0.050800	HA	0.136000	C5	0.078306	FE4	0.286432
CB	-0.201782	CB	-0.331615	H51	-0.027195	FE5	0.199194
HB3	0.062628	HB2	0.092141	H52	-0.027195	FE6	0.443866
HB2	0.062628	HB3	0.092141	C4	-0.102014	FE7	0.364096
SG	-0.617702	CG	0.049098	H41	-0.040309	FE8	0.679588
C	0.597300	ND1	0.333711	H42	-0.040309	C	0.080623
O	-0.567900	CE1	-0.155062	C3	0.567718	S1A	-0.579502
		HE1	0.176211	O7	-0.748105	S2A	-0.559513
		NE2	-0.261809	HO7	0.420578	S3A	-0.628324
		HE2	0.336131	C7	0.571617	S4A	-0.566862
		CD2	-0.362854	O5	-0.578050	S5A	-0.639053
		HD2	0.190886	O6	-0.673869	S1B	-0.674769
		C	0.597300	C2	-0.302704	S2B	-0.518351
		O	-0.567900	H21	0.021845	SH	0.173123
				H22	0.021845	S3B	-0.572746
				C1	0.718518	S4B	-0.571039
				O1	-0.725700		
				O2	-0.724461		

Table S7. RESP charges employed for the atoms in the P cluster (net charge -4).

	Cys-49	Cys-75	Cys-138	Cys-20B	Cys-45B	Cys-104B	Cluster	
N	-0.415700	-0.415700	-0.415700	-0.415700	-0.415700	-0.415700	FE1	0.557801
H	0.271900	0.271900	0.271900	0.271900	0.271900	0.271900	FE2	0.451139
CA	0.038025	0.038025	0.038025	0.038025	0.038025	0.038025	FE3	0.316013
HA	0.050800	0.050800	0.050800	0.050800	0.050800	0.050800	FE4	0.456648
CB	0.038086	0.025115	0.150718	0.041829	0.106944	0.227815	S1	-0.676365
HB3	-0.012282	-0.000132	-0.036995	-0.013831	-0.021215	-0.068997	S2A	-0.528870
HB2	-0.012282	-0.000132	-0.036995	-0.013831	-0.021215	-0.068997	S3A	-0.595334
SG	-0.666742	-0.508962	-0.654146	-0.677935	-0.499439	-0.612284	S4A	-0.682497
C	0.597300	0.597300	0.597300	0.597300	0.597300	0.597300	FE5	0.561976
O	-0.567900	-0.567900	-0.567900	-0.567900	-0.567900	-0.567900	FE6	0.336917
							FE7	0.269003
							FE8	0.481349
							S2B	-0.337973
							S3B	-0.578877
							S4B	-0.541572

Table S8. RESP charges employed for the atoms in the Mg site (net charge 0).

Glu-59B		Asp-301E		Mg and 4water	
N	-0.516300	N	-0.516300	Mg	1.146668
H	0.293600	H	0.293600	O	-0.783960
CA	0.039700	CA	0.339150	H	0.444145
HA	0.110500	HA	0.088000	H	0.444145
CB	0.357050	CB	-0.605155	O	-0.818987
HB2	0.158028	HB2	0.155355	H	0.418634
HB3	0.158028	HB3	0.155355	H	0.418634
CG	-0.620610	CG	0.938939	O	-0.844684
HG2	-0.042500	OD1	-0.664937	H	0.454144
HG3	-0.042500	OD2	-0.813399	H	0.454144
CD	0.930174	C	0.536600	O	-0.880370
OE1	-0.806477	O	-0.581900	H	0.436559
OE2	-0.624312			H	0.436559
C	0.536600				
O	-0.581900				

Table S9. Quality measures and Fe–O distances (*d* in Å) for the four quantum-refined structures with varying protonation states of homocitrate (cf. Figure 2) and different weights factors. In Phenix, $E_{\mathrm{tot}} = 0.5\, w_{\mathrm{xc}} E_{\mathrm{Xray}} + w_{\mathrm{c}} E_{\mathrm{MM}}$, so in practise $w_{\mathrm{A}} = \frac{0.5 w_{\mathrm{xc}}}{w_{\mathrm{c}}}$. The quality measures are the real-space Z-scores based on the difference maps (RSZD), the real-space *R* factors (RSR), the real-space correlation coefficients (RSCC) for homocitrate, the strain energy of the QM region (ΔE_{str}) and the sum of the difference in the Fe–O bond lengths ($\Sigma\Delta d$) to the homocitrate O5 (carboxylate) and O7 (alcohol) atoms in the quantum-refined structure (d(Fe–O)$_{\mathrm{QR}}$) and in a structure optimised by QM/MM without any crystallographic information (d(Fe–O)$_{\mathrm{QM}}$).

w_{xc}	w_{c}	HCA prot	RSZD	RSR	RSCC	ΔE_{str} (kJ/mol)	$\Sigma\Delta d$ (Å)	d(Fe–O)$_{\mathrm{QR}}$ O5	d(Fe–O)$_{\mathrm{QR}}$ O7	d(Fe–O)$_{\mathrm{QM}}$ O5	d(Fe–O)$_{\mathrm{QM}}$ O7
auto	1.0	H1c	0.6	0.043	0.968	168	0.17	2.26	2.18	2.22	2.05
		H1a	0.6	0.042	0.971	133	0.20	2.23	2.19	2.13	2.29
		2H	0.7	0.043	0.970	20	0.14	2.24	2.19	2.22	2.31
		0H	0.6	0.042	0.969	126	0.25	2.25	2.17	2.18	1.99
18.0	1.0	H1c	0.8	0.043	0.969	214	0.13	2.26	2.18	2.23	2.08
		H1a	0.6	0.042	0.971	159	0.22	2.23	2.18	2.12	2.29
		2H	0.6	0.043	0.970	156	0.15	2.25	2.18	2.22	2.30
		0H	0.8	0.043	0.967	172	0.25	2.26	2.16	2.18	1.99
2.0	1.0	H1c	1.7	0.050	0.955	54	0.08	2.23	2.13	2.22	2.06
		H1a	0.5	0.044	0.967	41	0.07	2.17	2.20	2.13	2.23
		2H	1.1	0.046	0.962	35	0.08	2.22	2.21	2.23	2.28
		0H	2.8	0.055	0.944	38	0.10	2.22	2.06	2.19	1.99
2.0	10.0	H1c	4.4	0.063	0.925	7	0.03	2.22	2.07	2.23	2.05
		H1a	1.5	0.049	0.958	3	0.00	2.13	2.25	2.13	2.25
		2H	2.6	0.051	0.950	1	0.01	2.21	2.28	2.21	2.29
		0H	5.0	0.054	0.945	0	0.00	2.16	1.99	2.16	1.99
18.0	10.0	H1c	2.3	0.052	0.951	41	0.08	2.23	2.12	2.23	2.04
		H1a	0.5	0.044	0.966	33	0.05	2.16	2.20	2.17	2.24
		2H	1.0	0.046	0.964	35	0.09	2.21	2.21	2.22	2.29
		0H	2.6	0.055	0.944	28	0.06	2.21	2.04	2.20	1.99

Table S10. Relative energies (ΔE in kJ/mol) and spin densities of the various BS states for the E_0 state of Fe-nitrogenase, calculated with r^{2}SCAN and TPSSh. BS is the number of the BS state in Nodleman's nomenclature.

State	BS	r^{2}SCAN									TPSSh								
		ΔE	Fe1	Fe2	Fe3	Fe4	Fe5	Fe6	Fe7	Fe8	ΔE	Fe1	Fe2	Fe3	Fe4	Fe5	Fe6	Fe7	Fe8
1234	1	123	-3.8	-3.5	-3.5	-3.4	3.6	3.5	3.5	3.9	121	-3.8	-3.6	-3.5	-3.4	3.6	3.5	3.5	3.8
1235	4	59	-3.5	-3.5	-3.5	3.5	-3.6	3.5	3.4	3.8	62	-3.6	-3.6	-3.5	3.6	-3.7	3.5	3.5	3.8
1236	5	80	-3.7	-3.5	-3.6	3.5	3.6	-3.6	3.5	3.8	81	-3.7	-3.5	-3.6	3.5	3.6	-3.6	3.6	3.8
1237	5	70	-3.7	-3.5	-3.6	3.5	3.5	3.6	-3.5	3.8	63	-3.7	-3.6	-3.6	3.5	3.6	3.6	-3.6	3.8
1238	3	97	-3.6	-3.4	-3.5	3.6	3.6	3.5	3.5	-3.9	92	-3.6	-3.6	-3.6	3.6	3.7	3.5	3.5	-3.7
1245	5	58	-3.7	-3.6	3.5	-3.5	-3.6	3.6	3.5	3.8	53	-3.7	-3.6	3.5	-3.5	-3.6	3.6	3.5	3.8
1246	5	63	-3.7	-3.5	3.5	-3.6	3.6	-3.6	3.6	3.8	57	-3.7	-3.6	3.5	-3.6	3.6	-3.6	3.6	3.8
1247	4	52	-3.7	-3.5	3.6	-3.4	3.5	3.5	-3.6	3.8	41	-3.7	-3.5	3.6	-3.5	3.5	3.5	-3.6	3.8
1248	3	84	-3.6	-3.5	3.5	-3.4	3.5	3.5	3.6	-3.9	81	-3.7	-3.6	3.6	-3.6	3.6	3.6	3.7	-3.7
1256	8	28	-3.6	-3.4	3.5	3.5	-3.6	-3.6	3.5	3.8	29	-3.6	-3.5	3.5	3.6	-3.6	-3.6	3.5	3.8
1257	7	3	-3.6	-3.5	3.4	3.4	-3.5	3.6	-3.5	3.8	0	-3.6	-3.6	3.4	3.5	-3.5	3.6	-3.5	3.7
1258	10	20	-3.6	-3.4	3.5	3.5	-3.5	3.6	3.6	-3.8	19	-3.6	-3.5	3.6	3.5	-3.5	3.6	3.6	-3.8
1267	8	28	-3.6	-3.4	3.5	3.5	3.5	-3.6	-3.6	3.8	29	-3.6	-3.5	3.5	3.5	3.6	-3.6	-3.6	3.8
1268	9	50	-3.6	-3.4	3.5	3.5	3.6	-3.5	3.6	-3.8	50	-3.6	-3.5	3.5	3.6	3.6	-3.5	3.6	-3.8
1278	10	23	-3.6	-3.5	3.5	3.5	3.6	3.6	-3.5	-3.8	22	-3.6	-3.5	3.5	3.6	3.6	3.6	-3.5	-3.8
1345	5	57	-3.7	3.5	-3.6	-3.4	-3.6	3.6	3.5	3.8	54	-3.7	3.5	-3.6	-3.5	-3.6	3.6	3.5	3.8
1346	4	38	-3.5	3.5	-3.5	-3.5	3.5	-3.6	3.4	3.8	42	-3.6	3.6	-3.5	-3.6	3.5	-3.7	3.5	3.8
1347	5	58	-3.6	3.5	-3.5	-3.6	3.5	3.6	-3.6	3.8	55	-3.7	3.5	-3.6	-3.6	3.6	3.6	-3.6	3.8
1348	3	75	-3.5	3.5	-3.4	-3.4	3.6	3.3	3.6	-3.9	87	-3.6	3.5	-3.5	-3.5	3.5	3.6	3.5	-3.7
1356	7	2	-3.6	3.4	-3.5	3.4	-3.5	-3.5	3.5	3.8	4	-3.6	3.4	-3.5	3.4	-3.5	-3.5	3.6	3.8
1357	8	32	-3.6	3.5	-3.4	3.5	-3.6	3.5	-3.6	3.8	33	-3.6	3.5	-3.5	3.5	-3.6	3.6	-3.6	3.8
1358	10	27	-3.6	3.5	-3.5	3.5	-3.5	3.6	3.6	-3.8	28	-3.6	3.6	-3.5	3.5	-3.5	3.6	3.6	-3.8
1367	8	23	-3.6	3.5	-3.4	3.5	3.5	-3.6	-3.6	3.8	26	-3.6	3.5	-3.5	3.5	3.6	-3.6	-3.6	3.8
1368	10	27	-3.6	3.5	-3.4	3.5	3.6	-3.5	3.6	-3.8	26	-3.6	3.5	-3.5	3.6	3.7	-3.5	3.6	-3.8
1378	9	49	-3.6	3.5	-3.5	3.5	3.6	3.6	-3.5	-3.8	53	-3.6	3.5	-3.5	3.5	3.6	3.6	-3.5	-3.8
1456	8	20	-3.6	3.5	3.5	-3.5	-3.6	-3.6	3.5	3.8	22	-3.6	3.6	3.5	-3.5	-3.6	-3.6	3.5	3.8
1457	8	29	-3.6	3.5	3.5	-3.5	-3.6	3.5	-3.6	3.8	29	-3.6	3.5	3.5	-3.5	-3.6	3.6	-3.6	3.7
1458	9	41	-3.6	3.5	3.5	-3.4	-3.5	3.6	3.6	-3.8	43	-3.6	3.6	3.6	-3.5	-3.6	3.6	3.6	-3.8
1467	7	0	-3.6	3.4	3.4	-3.5	3.6	-3.5	-3.5	3.8	1	-3.6	3.5	3.5	-3.6	3.6	-3.5	-3.5	3.8
1468	10	23	-3.6	3.5	3.5	-3.4	3.6	-3.5	3.6	-3.8	23	-3.6	3.5	3.6	-3.5	3.6	-3.6	3.6	-3.8
1478	10	27	-3.6	3.5	3.5	-3.5	3.6	3.6	-3.5	-3.8	29	-3.6	3.6	3.5	-3.5	3.6	3.6	-3.5	-3.8
1567	2	84	-3.3	3.3	3.4	3.4	-3.6	-3.5	-3.5	3.8	70	-3.4	3.5	3.5	3.5	-3.6	-3.5	-3.5	3.7
1568	6	57	-3.3	3.5	3.6	3.5	-3.5	-3.6	3.6	-3.8	48	-3.4	3.5	3.7	3.5	-3.6	-3.6	3.7	-3.8
1578	6	64	-3.3	3.6	3.5	3.5	-3.6	3.6	-3.6	-3.8	54	-3.4	3.7	3.5	3.5	-3.6	3.7	-3.6	-3.8
1678	6	67	-3.3	3.5	3.5	3.6	3.6	-3.6	-3.5	-3.8	58	-3.4	3.5	3.5	3.7	3.7	-3.6	-3.6	-3.8
2345	6	67	3.3	-3.5	-3.5	-3.4	-3.7	3.6	3.5	3.8	54	3.4	-3.6	-3.6	-3.5	-3.7	3.6	3.5	3.8
2346	6	63	3.3	-3.6	-3.5	-3.5	3.6	-3.6	3.6	3.8	47	3.4	-3.7	-3.5	-3.5	3.6	-3.7	3.6	3.8
2347	6	57	3.3	-3.5	-3.6	-3.5	3.5	3.6	-3.6	3.8	41	3.4	-3.5	-3.7	-3.5	3.6	3.6	-3.7	3.8
2348	2	82	3.3	-3.5	-3.4	-3.4	3.6	3.5	3.5	-3.8	59	3.4	-3.6	-3.5	-3.5	3.6	3.5	3.6	-3.7
2356	10	27	3.6	-3.5	-3.5	3.5	-3.6	-3.6	3.5	3.8	29	3.6	-3.6	-3.5	3.5	-3.6	-3.6	3.5	3.8
2357	10	23	3.6	-3.5	-3.5	3.4	-3.6	3.5	-3.6	3.8	18	3.6	-3.5	-3.6	3.5	-3.6	3.6	-3.6	3.8
2358	7	0	3.6	-3.4	-3.4	3.5	-3.6	3.5	3.5	-3.8	1	3.6	-3.5	-3.5	3.6	-3.6	3.5	3.5	-3.8
2367	9	41	3.6	-3.5	-3.5	3.4	3.5	-3.6	-3.6	3.8	43	3.6	-3.6	-3.6	3.5	3.6	-3.6	-3.6	3.8
2368	8	29	3.6	-3.5	-3.5	3.5	3.6	-3.5	3.6	-3.8	29	3.6	-3.5	-3.5	3.5	3.6	-3.6	3.6	-3.8
2378	8	21	3.6	-3.6	-3.5	3.5	3.6	3.6	-3.5	-3.8	16	3.6	-3.6	-3.6	3.5	3.6	3.6	-3.5	-3.8

2456	9	49	3.6	-3.5	3.5	-3.5	-3.6	-3.6	3.5	3.8	52	3.6	-3.5	3.5	-3.5	-3.6	-3.6	3.5	3.8
2457	10	27	3.6	-3.5	3.4	-3.5	-3.6	3.5	-3.6	3.8	26	3.6	-3.5	3.5	-3.6	-3.7	3.5	-3.6	3.8
2458	8	23	3.6	-3.5	3.4	-3.5	-3.5	3.6	3.6	-3.8	26	3.6	-3.5	3.5	-3.5	-3.6	3.6	3.6	-3.8
2467	10	27	3.6	-3.5	3.5	-3.5	3.5	-3.6	-3.6	3.8	28	3.6	-3.6	3.5	-3.5	3.5	-3.6	-3.6	3.8
2468	8	32	3.6	-3.5	3.4	-3.5	3.6	-3.5	3.6	-3.8	34	3.6	-3.5	3.5	-3.5	3.6	-3.6	3.6	-3.8
2478	7	2	3.6	-3.4	3.5	-3.4	3.5	3.5	-3.5	-3.8	4	3.6	-3.4	3.5	-3.4	3.5	3.5	-3.6	-3.8
2567	3	75	3.5	-3.5	3.4	3.4	-3.6	-3.3	-3.6	3.9	87	3.6	-3.5	3.5	3.5	-3.5	-3.6	-3.5	3.7
2568	5	61	3.7	-3.5	3.5	3.5	-3.5	-3.6	3.6	-3.8	55	3.7	-3.5	3.6	3.6	-3.6	-3.6	3.6	-3.8
2578	4	38	3.5	-3.5	3.5	3.5	-3.5	3.6	-3.4	-3.8	45	3.7	-3.6	3.5	3.5	-3.6	3.7	-3.5	-3.8
2678	5	57	3.7	-3.5	3.6	3.4	3.6	-3.6	-3.5	-3.8	62	3.7	-3.5	3.6	3.5	3.6	-3.6	-3.5	-3.8
3456	10	23	3.6	3.5	-3.5	-3.5	-3.6	-3.6	3.5	3.8	22	3.6	3.5	-3.5	-3.6	-3.6	-3.6	3.5	3.8
3457	9	50	3.6	3.4	-3.5	-3.5	-3.6	3.5	-3.6	3.8	50	3.6	3.5	-3.5	-3.6	-3.6	3.5	-3.6	3.8
3458	8	28	3.6	3.4	-3.5	-3.5	-3.5	3.6	3.6	-3.8	29	3.6	3.5	-3.5	-3.5	-3.6	3.6	3.6	-3.8
3467	10	20	3.6	3.4	-3.5	-3.5	3.5	-3.6	-3.6	3.8	19	3.6	3.5	-3.6	-3.5	3.5	-3.6	-3.6	3.8
3468	7	3	3.6	3.5	-3.4	-3.4	3.5	-3.6	3.5	-3.8	0	3.6	3.6	-3.4	-3.5	3.5	-3.6	3.5	-3.7
3478	8	28	3.6	3.4	-3.5	-3.5	3.6	3.6	-3.5	-3.8	29	3.6	3.5	-3.5	-3.6	3.6	3.6	-3.5	-3.8
3567	3	85	3.6	3.5	-3.6	3.4	-3.6	-3.5	-3.6	3.9	81	3.7	3.6	-3.6	3.6	-3.6	-3.6	-3.7	3.7
3568	4	50	3.7	3.5	-3.6	3.5	-3.4	-3.5	3.6	-3.8	41	3.7	3.5	-3.6	3.5	-3.5	-3.5	3.6	-3.8
3578	5	63	3.7	3.5	-3.5	3.6	-3.6	3.6	-3.6	-3.8	57	3.7	3.6	-3.5	3.6	-3.6	3.6	-3.6	-3.8
3678	5	58	3.7	3.6	-3.5	3.5	3.6	-3.6	-3.5	-3.8	53	3.7	3.6	-3.5	3.5	3.6	-3.6	-3.5	-3.8
4567	3	93	3.6	3.4	3.5	-3.6	-3.6	-3.5	-3.5	3.9	92	3.6	3.6	3.6	-3.6	-3.7	-3.5	-3.5	3.7
4568	5	70	3.7	3.5	3.6	-3.5	-3.5	-3.6	3.5	-3.8	62	3.7	3.6	3.6	-3.5	-3.6	-3.6	3.6	-3.8
4578	5	80	3.7	3.5	3.6	-3.5	-3.6	3.6	-3.5	-3.8	74	3.7	3.6	3.6	-3.6	-3.6	3.6	-3.6	-3.8
4678	4	60	3.5	3.5	3.5	-3.5	3.6	-3.5	-3.4	-3.8	62	3.7	3.5	3.5	-3.6	3.6	-3.5	-3.5	-3.8
5678	1	123	3.8	3.5	3.5	3.4	-3.6	-3.5	-3.5	-3.9	121	3.8	3.6	3.5	3.5	-3.6	-3.5	-3.5	-3.8

Table S11. Hydrogen bonds around the studied residues in the 0H+HIE MD simulation. The table show the percentage of the snapshots in which the hydrogen bond is observed (occ) and the average distance in those snapshots (dist in Å).

atom1		atom2		A		D	
				occ	distance	occ	distance
N	Gln-176	H	Ser-177	41	2.41	53	2.40
OE1		H	Gly-48	56	2.22	97	2.02
		H	Gln-176	99	2.03	92	2.10
		HE22	Gln-176	81	2.39	83	2.39
O		H	Gly-179	73	2.20	94	2.07
		H	His-180	83	2.11	45	2.26
N	His-180	H	His-181	18	2.43	24	2.42
ND1		H	His-180			42	2.33
		H1	WAT	89	2.03	67	2.08
NE2		H1	WAT			14	2.37
O		H	Asn-184	78	2.24	82	2.23
O		HD21	Asn-184	98	1.95	98	1.93
O1	HCA	HE2	His-56			51	1.98
		H1	WAT	257	1.95	212	1.92
O2		HE2	His-56	79	1.92	36	2.00
		HZ	Lys-83	19	2.01	13	1.99
		H1	WAT	170	1.94	251	1.89
O3		H	Lys-406	96	1.97	97	2.01
		H1	WAT	286	1.93	276	1.89
O4		H1	WAT	294	1.86	292	1.90
O5		HZ	Lys-83	57	2.29	56	2.31
		H1	WAT	100	1.84	100	1.82
O6		HZ	Lys-83	28	2.12	35	2.16
		H1	WAT	209	1.94	194	1.91
O7		HZ	Lys-361	88	1.99	100	1.97
		H1	WAT	24	1.83		
S1A	FFC	H	Ser-338	100	2.28	100	2.35
		HG	Ser-338	12	2.73		
		H1	WAT	96	2.17	96	2.20
S2A		HE2	His-180			15	2.77
		H1	WAT			26	2.63
S3A		H	Gly-336	18	2.81	51	2.73
		H	Gly-337	100	2.37	100	2.26
		H	Lys-339	98	2.51	87	2.74
		HZ	Lys-339	56	2.65	62	2.61
S4A		HG	Ser-338			16	2.63
		HZ	Lys-339	103	2.58	93	5.52

	H2	WAT	210	2.35	190	2.42
S5A	HZ	Lys-83	20	2.66		
	H1	WAT	268	2.27	291	2.26
S1B	H	Gly-336	14	2.87		
	HZ	Lys-361	34	2.82	63	2.74
S2B	HE2	His-180	81	2.54	90	2.49
	H1	WAT	208	2.31	231	2.32
S3B	HZ	Lys-83	123	2.30	129	2.34
	H1	WAT	100	2.41	79	2.68
S4B	HZ	Lys-339	117	2.33	122	2.38
	H1	WAT	18	2.80	28	2.77

Table S12. Hydrogen bonds around the studied residues in the 1Ha+HID MD simulation. The table show the percentage of the snapshots in which the hydrogen bond is observed (occ) and the average distance in those snapshots (dist in Å).

atom1		atom2		A		D	
				occ	dist	occ	dist
N	Gln-176	H	Ser-77	48	2.40	49	2.38
OE1		H	Gly-48	52	2.04		
		HG	Cys-52	10	2.21	46	2.07
		HE22	Gln-176	89	2.37	93	2.36
		H1	WAT	91	1.89	127	1.91
O		H	Gly-179	85	2.14	69	2.23
		H	His-180	30	2.28	84	2.13
N	His-180	H	His-181	26	2.42	19	2.43
		H1	WAT	50	2.02		
O		H	Asn-184	84	2.23	88	2.19
		HD21	Asn-184	98	1.98	93	2.01
O1	HCA	HE2	His-56	47	1.92		
		HE22	Gln-176			97	1.93
		HZ	Lys-361			100	1.79
		HO7	HCA			80	2.13
		H1	WAT	173	2.11		
O2		HE2	His-56	52	1.88		
		H2	WAT	178	1.91	305	1.89
O3		HZ	Lys-361	52	2.13	73	2.07
		H	Lys-406			229	1.84
		HO7	HCA	17	1.95		
		H1	WAT	204	2.44		
O4		HZ	Lys-361	60	2.12		
		H	Lys-406	12	2.26	64	1.94
		H1	WAT	195	1.98	200	1.87
O5		HZ	Lys-83			24	2.36
		H1	WAT	103	1.95	102	1.83
O6		HZ	Lys-83			71	2.02
		H1	WAT	246	1.99	195	1.90
O7		H1	WAT	39	1.86		
S1A	FFC	H	Ser-338	100	2.22	100	2.16
		HG	Ser-338	39	2.70	85	2.45
		H2	WAT	104	2.22	46	2.45
S2A		H1	WAT	42	2.66	11	2.55
S3A		H	Gly-336	10	2.81		
		H	Gly-337	99	2.47	99	2.48
		H	Lys-339	98	2.57	100	2.41

	HZ	Lys-339	21	2.75		
S4A	HZ	Lys-339	63	2.55	60	2.52
	H1	WAT	188	2.44	191	2.30
S5A	HZ	Lys-83	49	2.62		
	HZ	Lys-339			11	2.84
	H1	WAT	194	2.28	252	2.28
S1B	H	Gly-336	11	2.89		
	H1	WAT	60	2.42		
S2B	HE21	Gln-176	28	2.58	97	2.27
	H1	WAT	197	2.38	54	2.25
S3B	HZ	Lys-83	113	2.20	133	2.39
	HO7	HCA	30	2.75		
	H1	WAT	86	2.46		
S4B	HZ	Lys-339	107	2.23	105	2.18
	H1	WAT	52	2.76	35	2.81

Table S13. Hydrogen bonds around the studied residues in the 1Ha+HIE MD simulation. The table show the percentage of the snapshots in which the hydrogen bond is observed (occ) and the average distance in those snapshots (dist in Å).

atom1		atom2		A		D	
				occ	dist	occ	dist
N	Gln-176	H	Ser-176	31	2.41	40	2.41
OE1		H	Gln-175	99	2.00	92	2.13
		H	Gly-47	84	2.39	100	1.97
		HE22	Gln-175	45	2.24	86	2.38
NE2		HG	Cys-51	13	2.31		
O		H	Gly-178	95	2.10	83	2.15
		H	His-179	73	2.22	61	2.13
N	His-180	H	His-181	15	2.43	21	2.43
ND1		H	His-180			23	2.29
		H2	WAT	54	2.01	71	2.04
O		H	Asn-184	77	2.25	84	2.21
		HD21	Asn-184	91	2.00	93	1.98
O1	HCA	HE21	Gln-176	20	2.14	94	2.05
		HZ	Lys-361	19	2.03	98	2.09
		HO7	HCA	99	1.85	99	1.85
		H1	WAT	164	1.93	26	2.26
O2		HG	Cys-52	20	2.26	20	2.29
		HE21	Gln-176			12	2.25
		H1	WAT	276	1.93	261	1.85
O3		H	Lys-406	67	2.03	50	1.99
		H1	WAT	261	1.87	270	1.89
O4		H	Lys-406	47	2.05	46	2.03
		H1	WAT	263	1.90	286	1.91
O5		HZ	Lys-83	24	2.34	18	2.34
		H1	WAT	100	1.82	101	1.82
O6		HZ	Lys-83	13	2.21		
		H1	WAT	195	1.90	204	1.87
O7		HZ	Lys-361			53	2.29
		H1	WAT	16	2.26		
S1A	FFC	H	Ser-338	100	2.27	100	2.31
		HG	Ser-338	67	2.48	23	2.70
		H1	WAT	59	2.25	45	2.23
S2A		HE2	His-180			10	2.83
		HG	Ser-260	24	2.41		
		H1	WAT			15	2.56
S3A		H	Gly-336			36	2.74

	H	Gly-337	99	2.55	100	2.30
	H	Lys-339	100	2.39	83	2.71
	HZ	Lys-339	26	2.78	10	2.82
S4A	HG	Ser-338			21	2.51
	HZ	Lys-339	95	2.51	53	2.53
	H1	WAT	166	2.43	178	2.49
S5A	HZ	Lys-83			12	2.63
	HZ	Lys-339	11	2.90		
	H1	WAT	210	2.20	208	2.22
S1B	HZ	Lys-361			43	2.60
	HO7	HCA			30	2.91
	H1	WAT	26	2.25		
S2B	HE2	His-180	88	2.37	91	2.45
	H1	WAT	154	2.29	70	2.38
S3B	HZ	Lys-83	106	2.14	112	2.19
	HO7	HCA			19	2.82
	H2	WAT	82	2.43		
S4B	HZ	Lys-			103	2.19
	H1	WAT			22	2.83

Table S14. Hydrogen bonds around the studied residues in the 1Ha+HIP MD simulation. The table show the percentage of the snapshots in which the hydrogen bond is observed (occ) and the average distance in those snapshots (dist in Å).

atom1		atom2		A		D	
				occ	dist	occ	dist
N	Gln-176	H	Ser-77	41	2.41	44	2.41
OE1		H	Gly-48	89	2.14	92	2.13
		H	Gln-176	98	2.03	98	2.02
		HE22	Gln-176	83	2.39	82	2.39
NE2		H	Gly-48	13	2.37	9	2.39
O		H	Gly-179	53	2.28	56	2.26
		H	His-180	90	2.14	77	2.16
N	His-180	H	His-181	13	2.44	15	2.43
		H	Asn-184	63	2.29	49	2.32
		HD21	Asn-184	96	1.96	96	1.96
S1A	FFC	H	Ser-338	98	2.47	100	2.31
		HG	Ser-338	37	2.62	25	2.63
S3A		H	Gly-337	99	2.51	100	2.45
		H	Lys-339	99	2.56	96	2.62
		HZ	Lys-339	37	2.67	37	2.73
S4A		HG	Ser-338	3	2.89	12	2.72
		HZ	Lys-339	62	2.61	91	2.58
		H1	WAT	214	2.18	172	2.37
S5A		HZ	Lys-83	3	2.82	18	2.83
		H1	WAT	242	2.77	226	2.29
		HZ	Lys-361	2	2.84	11	2.78
S1B		HO7	HCA	4	2.86	29	2.91
		H1	WAT	64	2.23	1	2.64
S2B		HE2	His-180	95	2.44	97	2.34
		H1	WAT	108	2.65	52	2.24
S3B		HZ	Lys-83	110	2.19	115	2.19
		HO7	HCA	4	2.24	23	2.84
S4B		HZ	Lys-339	105	2.67	114	2.30
		H1	WAT	23	2.73	35	2.79

Table S15. Hydrogen bonds around the studied residues in the 1Hc+HIE MD simulation. The table show the percentage of the snapshots in which the hydrogen bond is observed (occ) and the average distance in those snapshots (dist in Å).

atom1		atom2		A		D	
				occ	dist	occ	dist
N	Gln-176	H	Ser-77	52	2.40	34	2.41
OE1		H	Gly-48	82	2.02	89	2.13
		H	Gln-176	64	2.15	98	2.04
		HE22	Gln-176	85	2.38	82	2.39
		H1	WAT	27	1.90		
O		H	Gly-179	72	2.22	78	2.21
		H	His-180	77	2.20	96	2.07
N	His-180	H	His-181	23	2.43	17	2.44
		H	Asn-184	67	2.26	77	2.28
ND2		HD21	Asn-184	99	1.91	98	1.95
O		H1	WAT	58	2.10	37	1.93
		H1	WAT	17	2.32		
O1	HCA	HE2	His-56	48	2.00	63	1.99
		HO2	HCA	12	2.11	56	2.10
		H1	WAT	146	2.00	112	1.96
O2		HE2	His-56			10	2.18
		HE21	Gln-176	14	2.31	17	2.18
		HZ	Lys-361	14	2.33		
		H1	WAT	73	2.14	65	2.08
O3		HZ	Lys-361	20	1.91		
		H	Lys-406	37	1.99	80	1.98
		H1	WAT	255	1.87	255	1.89
O4		H	Lys-406	26	2.04	274	1.91
		HO2	HCA	24	1.84		
		H1	WAT	265	1.91		
O5		HZ	Lys-83	22	2.32		
		H1	WAT	104	1.87	103	1.87
O6		HZ	Lys-83	10	2.21		
		H1	WAT	191	1.89	206	1.88
O7		HZ	Lys-361	82	2.01	94	2.01
		H1	WAT	31	1.93	10	1.91
S1A	FFC	H	Ser-337	100	2.30	100	2.36
		HG	Ser-337	52	2.54		
S3A		H	Gly-335			46	2.76
		H	Gly-336	100	2.43	99	2.33
		H	Lys-338	99	2.50	76	2.78
		HZ	Lys-338	31	2.78	28	2.78

S4A	HZ	Lys-338	105	2.50	101	2.47
	H2	WAT	165	2.40	211	2.52
S5A	HZ	Lys-82	34	2.73	101	2.52
	HZ	Lys-338	13	2.89		
	H1	WAT	205	2.26	172	2.32
S1B	HZ	Lys-360	37	2.69	74	2.71
	H1	WAT	36	2.50	19	2.66
S2B	HE21	Gln-175	18	2.21		
	HE2	His-179	91	2.46	99	2.32
	H1	WAT	146	2.37	107	2.27
S3B	HZ	Lys-82	106	2.13	107	2.17
	H1	WAT	86	2.53	91	2.51
S4B	HZ	Lys-338	108	2.24		
	H1	WAT	27	2.81		

Table S16. Hydrogen bonds around the studied residues in the 2H+HIE MD simulation. The table show the percentage of the snapshots in which the hydrogen bond is observed (occ) and the average distance in those snapshots (dist in Å).

atom1		atom2		A		D	
				occ	dist	occ	dist
N	Gln-176	H	Ser-77	50	2.40	39	2.41
OE1		H	Gln-176	98	2.03	99	2.05
		H	Gly-48	89	2.16	92	2.12
		HE22	Gln-176	86	2.38	83	2.38
NE2		H1	WAT	16	2.34		
O		H	His-180	94	2.11	93	2.11
		H	Gly-179	78	2.23	76	2.22
N	His-180	H	His-181	15	2.43	16	2.43
		H	Asn-184	86	2.24	68	2.26
		HD21	Asn-184	93	2.00	95	1.96
ND1		H1	WAT	97	1.96	42	1.98
O1	HCA	HE21	Gln-176	30	2.23		
		HO7	HCA	32	2.20		
		HG	Cys-52	11	2.33		
		HO2	HCA			100	2.11
		H1	WAT	94	2.10	69	2.17
O2		HE21	Gln-176	48	2.12	73	2.10
		HG	Cys-52	14	2.29		
		H1	WAT	53	2.16		
O3		HZ	Lys-361			54	1.90
		H	Lys-406	23	2.09		
		HO7	HCA	10	1.81		
		H1	WAT	287	1.90	218	1.89
O4		HZ	Lys-361			62	1.91
		H	Lys-406	30	2.08		
		H1	WAT	268	1.91	212	1.90
O5		H1	WAT	102	1.86	99	1.84
O6		H1	WAT	193	1.89	206	1.89
O7		H1	WAT			12	2.35
S1A	FFC	H	Ser-338	100	2.32	100	2.29
		HG	Ser-338	44	2.67	38	2.69
S2A		HG	Ser-260	24	2.56		
S3A		H	Gly-337	96	2.59	99	2.50
		H	Lys-339	100	2.50	90	2.73
		HZ	Lys-339	26	2.80	44	2.76
S4A		HG	Ser-338	11	2.82	19	2.83
		HZ	Lys-339	81	2.55	91	2.57

	H1	WAT	221	2.60	210	2.59
S5A	HZ	Lys-83	29	2.67	99	2.49
	H2	WAT	201	2.29	179	2.38
S1B	H	Gly-336	16	2.89		
	H1	WAT	100	2.22	86	2.44
S2B	HE2	His-180	97	2.47	98	2.50
	H2	WAT	93	2.36	107	2.39
S3B	HO7	HCA			50	2.47
	HO2	HCA	20	2.59		
	HZ	Lys-83	106	2.22	110	2.17
	H1	WAT	64	2.42	31	2.63
S4B	HZ	Lys-339	102	2.26	103	2.36
	H1	WAT	16	2.85	95	2.72

Table S17. Relative energies (ΔE in kJ/mol) and spin densities of the various BS states for the E_1 state of Fe-nitrogenase, calculated with r^2SCAN and TPSSh with the minimal QM system using the S2B(3) protonation state.

BS	r^2SCAN									TPSSh								
	ΔE	Fe1	Fe2	Fe3	Fe4	Fe5	Fe6	Fe7	Fe8	ΔE	Fe1	Fe2	Fe3	Fe4	Fe5	Fe6	Fe7	Fe8
1234	83	-3.7	-3.3	-3.4	-3.4	3.6	3.5	3.5	3.9	103	-3.6	-3.5	-3.5	-3.5	3.7	3.6	3.6	3.9
5678	92	3.8	3.5	3.7	3.5	-3.4	-3.2	-3.5	-3.9	91	3.8	3.5	3.7	3.6	-3.5	-3.3	-3.5	-3.9
1258	27	-3.6	-3.1	3.6	3.5	-3.3	3.6	3.6	-3.8	20	-3.6	-3.3	3.6	3.6	-3.4	3.6	3.6	-3.8
3467	11	3.6	3.4	-3.4	-3.4	3.5	-3.5	-3.5	3.8	16	3.7	3.5	-3.4	-3.4	3.6	-3.5	-3.5	3.8
1278	35	-3.5	-3.2	3.6	3.6	3.6	3.6	-3.3	-3.8	30	-3.6	-3.3	3.6	3.6	3.7	3.6	-3.4	-3.8
3456	15	3.6	3.4	-3.4	-3.3	-3.5	-3.5	3.5	3.8	23	3.7	3.5	-3.4	-3.4	-3.5	-3.5	3.6	3.8
1358	36	-3.5	3.5	-3.2	3.5	-3.3	3.6	3.6	-3.8	29	-3.5	3.5	-3.3	3.6	-3.4	3.6	3.6	-3.8
2467	0	3.6	-3.3	3.5	-3.4	3.5	-3.5	-3.6	3.8	4	3.6	-3.3	3.6	-3.5	3.5	-3.5	-3.6	3.8
1368	41	-3.5	3.5	-3.3	3.5	3.7	-3.1	3.6	-3.8	35	-3.6	3.5	-3.4	3.6	3.7	-3.2	3.6	-3.7
2457	25	3.6	-3.4	3.5	-3.3	-3.5	3.4	-3.5	3.8	33	3.6	-3.4	3.6	-3.5	-3.5	3.5	-3.5	3.8
1468	34	-3.6	3.5	3.5	-3.3	3.7	-3.2	3.7	-3.8	27	-3.6	3.5	3.6	-3.4	3.7	-3.3	3.7	-3.8
2357	24	3.6	-3.4	-3.4	3.5	-3.5	3.4	-3.5	3.8	34	3.6	-3.2	-3.5	3.6	-3.6	3.5	-3.6	3.8
1478	38	-3.1	3.6	3.6	-3.4	3.6	3.5	-3.5	-3.8	35	-3.3	3.6	3.6	-3.4	3.6	3.6	-3.5	-3.8
2356	3	3.6	-3.3	-3.5	3.5	-3.5	-3.4	3.5	3.8	10	3.6	-3.4	-3.5	3.5	-3.6	-3.5	3.6	3.8
1567	51	-3.3	3.5	3.6	3.5	-3.5	-3.2	-3.5	3.7	35	-3.4	3.5	3.6	3.6	-3.5	-3.3	-3.5	3.7
2348	53	3.3	-3.1	-3.4	-3.4	3.6	3.5	3.6	-3.7	39	3.4	-3.2	-3.4	-3.4	3.7	3.6	3.6	-3.7
1238	40	-3.6	-3.4	-3.4	3.6	3.7	3.6	3.6	-3.7	37	-3.6	-3.4	-3.5	3.6	3.7	3.6	3.6	-3.7
4567	28	3.6	3.4	3.5	-3.5	-3.4	-3.3	-3.4	3.9	43	3.6	3.5	3.5	-3.5	-3.4	-3.3	-3.4	3.8
1248	33	-3.5	-3.4	3.6	-3.4	3.6	3.6	3.7	-3.7	31	-3.6	-3.4	3.6	-3.5	3.6	3.6	3.7	-3.7
3567	23	3.5	3.4	-3.5	3.5	-3.4	-3.3	-3.4	3.9	39	3.6	3.5	-3.5	3.5	-3.4	-3.3	-3.4	3.7
1348	35	-3.5	3.6	-3.4	-3.4	3.7	3.5	3.6	-3.7	36	-3.5	3.6	-3.4	-3.4	3.7	3.5	3.7	-3.7
2567	40	3.5	-3.5	3.5	3.4	-3.4	-3.4	-3.4	3.9	64	3.6	-3.0	3.5	3.5	-3.5	-3.4	-3.5	3.7
1346	42	-3.5	3.5	-3.5	-3.5	3.5	-3.3	3.5	3.8	55	-3.6	3.3	-3.5	-3.5	3.7	-3.3	3.7	3.8
2578	47	3.5	-3.1	3.6	3.6	-3.5	3.6	-3.4	-3.8	42	3.6	-3.2	3.6	3.6	-3.5	3.6	-3.5	-3.8
1247	3	-3.5	-3.3	3.6	-3.4	3.5	3.5	-3.6	3.8	13	-3.5	-3.4	3.6	-3.4	3.6	3.6	-3.6	3.8
3568	39	3.6	3.5	-3.2	3.6	-3.4	-3.4	3.7	-3.8	35	3.6	3.6	-3.3	3.6	-3.5	-3.5	3.7	-3.8
1235	17	-3.5	-3.3	-3.4	3.6	-3.6	3.5	3.5	3.8	27	-3.6	-3.4	-3.4	3.6	-3.6	3.6	3.6	3.8
4678	50	3.7	3.5	3.6	-3.3	3.7	-3.5	-3.4	-3.8	42	3.7	3.5	3.6	-3.3	3.7	-3.5	-3.5	-3.8
1347	17	-3.5	3.5	-3.4	-3.4	3.6	3.5	-3.5	3.8	29	-3.5	3.5	-3.4	-3.5	3.7	3.6	-3.6	3.8
2568	44	3.7	-3.4	3.6	3.6	-3.5	-3.3	3.6	-3.8	37	3.7	-3.5	3.7	3.6	-3.5	-3.4	3.6	-3.8
1345	22	-3.5	3.5	-3.4	-3.4	-3.5	3.5	3.6	3.8	33	-3.5	3.5	-3.5	-3.4	-3.6	3.6	3.7	3.8
2678	38	3.6	-3.4	3.6	3.5	3.7	-3.3	-3.4	-3.8	43	3.7	-3.5	3.6	3.6	3.7	-3.3	-3.5	-3.8
1246	6	-3.6	-3.4	3.5	-3.5	3.6	-3.5	3.6	3.8	16	-3.6	-3.4	3.6	-3.5	3.7	-3.5	3.6	3.8
3578	43	3.7	3.5	-3.4	3.6	-3.4	3.6	-3.3	-3.7	40	3.7	3.5	-3.4	3.7	-3.4	3.6	-3.3	-3.8
1245	11	-3.5	-3.4	3.6	-3.4	-3.6	3.6	3.5	3.8	25	-3.6	-3.4	3.6	-3.4	-3.6	3.6	3.6	3.8
3678	48	3.6	3.6	-3.5	3.5	3.7	-3.3	-3.3	-3.8	54	3.7	3.6	-3.5	3.6	3.7	-3.4	-3.4	-3.8
1237	20	-3.6	-3.4	-3.4	3.6	3.5	3.6	-3.6	3.8	31	-3.6	-3.4	-3.4	3.6	3.6	3.6	-3.6	3.8
4568	66	3.6	3.6	3.5	-3.4	-3.5	-3.2	3.7	-3.8	71	3.6	3.6	3.6	-3.4	-3.5	-3.3	3.7	-3.8
1236	17	-3.6	-3.4	-3.5	3.5	3.6	-3.5	3.6	3.8	25	-3.6	-3.4	-3.5	3.6	3.6	-3.5	3.7	3.8

4578	57	3.7	3.5	3.6	-3.5	-3.3	3.6	-3.3	-3.7	62	3.7	3.5	3.7	-3.5	-3.3	3.6	-3.4	-3.8
1568	28	-3.3	3.5	3.6	3.6	-3.3	-3.3	3.7	-3.8	20	-3.4	3.6	3.7	3.6	-3.4	-3.4	3.7	-3.8
2347	27	3.3	-3.3	-3.4	-3.3	3.6	3.5	-3.6	3.8	27	3.4	-3.3	-3.4	-3.3	3.6	3.6	-3.6	3.8
1578	44	-3.3	3.4	3.6	3.6	-3.4	3.6	-3.3	-3.7	49	-3.4	3.5	3.6	3.6	-3.4	3.6	-3.4	-3.7
2346	21	3.3	-3.4	-3.4	-3.4	3.6	-3.6	3.6	3.8	20	3.4	-3.4	-3.4	-3.4	3.6	-3.6	3.6	3.8
1678	34	-3.3	3.5	3.6	3.6	3.7	-3.3	-3.3	-3.8	26	-3.4	3.6	3.6	3.7	3.7	-3.4	-3.4	-3.8
2345	33	3.4	-3.3	-3.4	-3.4	-3.6	3.5	3.6	3.8	40	3.7	-3.3	-3.4	-3.4	-3.7	3.4	3.4	3.8
1467	18	-3.6	3.5	3.5	-3.3	3.7	-3.3	-3.5	3.8	13	-3.3	3.5	3.5	-3.5	3.6	-3.5	-3.6	3.8
2358	14	3.6	-3.0	-3.4	3.6	-3.5	3.5	3.5	-3.8	11	3.6	-3.1	-3.4	3.6	-3.6	3.5	3.6	-3.8
1356	16	-3.6	3.4	-3.4	3.5	-3.4	-3.1	3.6	3.8	16	-3.6	3.5	-3.5	3.5	-3.5	-3.2	3.7	3.8
2478	13	3.6	-3.0	3.5	-3.4	3.5	3.5	-3.5	-3.8	12	3.6	-3.1	3.6	-3.4	3.5	3.5	-3.6	-3.8
1257	20	-3.6	-3.2	3.5	3.5	-3.4	3.6	-3.5	3.8	23	-3.4	-3.4	3.5	3.5	-3.5	3.6	-3.5	3.8
3468	13	3.6	3.5	-3.4	-3.4	3.5	-3.2	3.6	-3.8	4	3.7	3.5	-3.4	-3.4	3.6	-3.3	3.6	-3.7
1457	37	-3.6	3.5	3.6	-3.2	-3.5	3.5	-3.4	3.8	40	-3.2	3.5	3.6	-3.4	-3.6	3.5	-3.6	3.8
2368	14	3.6	-3.2	-3.4	3.5	3.6	-3.4	3.6	-3.8	12	3.6	-3.3	-3.5	3.6	3.6	-3.5	3.7	-3.8
1357	37	-3.5	3.5	-3.3	3.6	-3.4	3.5	-3.5	3.8	40	-3.5	3.5	-3.3	3.6	-3.5	3.6	-3.6	3.8
2468	8	3.6	-3.2	3.5	-3.5	3.6	-3.4	3.6	-3.8	8	3.6	-3.3	3.6	-3.5	3.7	-3.5	3.6	-3.8
1456	31	-3.6	3.5	3.5	-3.4	-3.5	-3.3	3.5	3.8	45	-3.3	3.6	3.6	-3.5	-3.6	-3.5	3.6	3.8
2378	23	3.6	-3.4	-3.3	3.5	3.6	3.6	-3.4	-3.8	19	3.6	-3.4	-3.4	3.6	3.6	3.6	-3.5	-3.8
1256	9	-3.6	-3.4	3.5	3.6	-3.5	-3.2	3.5	3.8	6	-3.6	-3.4	3.6	3.6	-3.5	-3.3	3.6	3.8
3478	14	3.6	3.5	-3.2	-3.4	3.6	3.6	-3.4	-3.8	12	3.7	3.5	-3.3	-3.4	3.7	3.6	-3.5	-3.8
1367	37	-3.5	3.5	-3.3	3.5	3.6	-3.2	-3.5	3.8	34	-3.6	3.5	-3.4	3.5	3.6	-3.3	-3.6	3.8
2458	25	3.6	-3.2	3.5	-3.4	-3.4	3.6	3.6	-3.8	21	3.6	-3.3	3.6	-3.5	-3.5	3.6	3.6	-3.8
1267	11	-3.6	-3.4	3.5	3.5	3.5	-3.2	-3.5	3.8	2	-3.7	-3.5	3.6	3.6	3.6	-3.3	-3.5	3.8
3458	29	3.6	3.5	-3.2	-3.5	-3.4	3.5	3.6	-3.8	24	3.7	3.5	-3.3	-3.5	-3.5	3.6	3.7	-3.8
1268	9	-3.6	-3.3	3.6	3.6	3.7	-3.3	3.7	-3.8	0	-3.6	-3.4	3.6	3.6	3.7	-3.4	3.7	-3.8
3457	27	3.6	3.4	-3.4	-3.3	-3.5	3.5	-3.5	3.8	42	3.6	3.5	-3.4	-3.4	-3.5	3.5	-3.5	3.8
1378	39	-3.5	3.5	-3.2	3.6	3.6	3.6	-3.3	-3.7	38	-3.5	3.5	-3.3	3.6	3.7	3.6	-3.4	-3.7
2456	19	3.6	-3.3	3.5	-3.4	-3.5	-3.4	3.5	3.8	30	3.6	-3.3	3.5	-3.4	-3.5	-3.5	3.5	3.8
1458	41	-3.5	3.5	3.6	-3.2	-3.3	3.6	3.6	-3.8	37	-3.6	3.6	3.6	-3.3	-3.4	3.6	3.7	-3.7
2367	15	3.6	-3.4	-3.4	3.4	3.5	-3.4	-3.5	3.8	25	3.6	-3.4	-3.4	3.5	3.5	-3.5	-3.5	3.8

Table S18. Relative energies (ΔE in kJ/mol) and spin densities of the various protonation states for the E_1 state of Fe-nitrogenase, calculated with r^{2}SCAN and TPSSh with the large QM system.

Protonated	r^{2}SCAN									TPSSh								
atom	ΔE	Fe1	Fe2	Fe3	Fe4	Fe5	Fe6	Fe7	Fe8	ΔE	Fe1	Fe2	Fe3	Fe4	Fe5	Fe6	Fe7	Fe8
Cys-257	126	3.5	-3.4	3.5	-3.2	3.6	-3.3	3.5	-3.7	123	3.5	-3.4	3.5	-3.3	3.6	-3.4	3.6	-3.7
His-423	107	3.6	-3.2	3.5	-3.4	3.6	-3.4	3.6	-3.8	109	3.6	-3.2	3.5	-3.4	3.6	-3.5	3.6	-3.7
HCA	184	3.6	-3.4	3.4	-3.5	3.5	-3.4	3.5	-3.8	205	3.6	-3.5	3.5	-3.5	3.6	-3.5	3.6	-3.7
C2367	77	3.6	-3.5	3.6	-3.3	3.6	-3.3	3.6	-3.8	77	3.6	-3.5	3.6	-3.3	3.6	-3.5	3.6	-3.7
C2456	76	3.6	-3.5	3.5	-3.4	3.7	-3.5	3.6	-3.8	50	3.6	-3.5	3.6	-3.5	3.7	-3.6	3.7	-3.7
C3457	51	3.6	-3.3	3.6	-3.5	3.7	-3.4	3.6	-3.8	33	3.6	-3.4	3.6	-3.5	3.7	-3.5	3.7	-3.7
S1A(Fe1)	83	3.6	-3.1	3.4	-3.4	3.6	-3.4	3.6	-3.8	78	3.6	-3.1	3.5	-3.4	3.6	-3.5	3.6	-3.7
S1A(Fe4)	75	3.6	-3.4	3.4	-3.0	3.6	-3.4	3.5	-3.8	78	3.6	-3.5	3.5	-3.2	3.6	-3.5	3.6	-3.7
S1B(Mo)	88	3.6	-3.4	3.5	-3.4	3.5	-3.2	3.5	-3.7	82	3.6	-3.5	3.6	-3.4	3.5	-3.3	3.5	-3.7
S1B(Fe6)	61	3.6	-3.4	3.5	-3.4	3.5	-3.0	3.5	-3.7	62	3.6	-3.4	3.6	-3.4	3.5	-3.2	3.6	-3.7
S2A(Fe1)	51	3.5	-3.2	3.5	-3.3	3.6	-3.4	3.6	-3.8	53	3.6	-3.2	3.5	-3.4	3.6	3.6	3.6	-3.7
S2A(Mo)	51	3.6	-3.1	3.4	-3.3	3.6	-3.4	3.6	-3.8	53	3.6	-3.2	3.5	-3.4	3.6	-3.5	3.6	-3.7
S3B(Fe6)	62	3.6	-3.4	3.5	-3.4	3.5	-3.0	3.5	-3.7	65	3.6	-3.4	3.5	-3.4	3.6	-3.1	3.6	-3.7
S3B(Mo)	76	3.6	-3.4	3.5	-3.4	3.5	-3.1	3.5	-3.6	73	3.6	-3.4	3.5	-3.4	3.5	-3.2	3.6	-3.7
S4A(Fe1)	57	3.6	-3.4	3.5	-3.0	3.5	-3.4	3.5	-3.8	39	3.6	-3.4	3.5	-3.1	3.6	-3.5	3.6	-3.7
S4A(Mo)	63	3.6	-3.4	3.4	-3.1	3.6	-3.3	3.6	-3.8	60	3.6	-3.4	3.5	-3.2	3.6	-3.4	3.6	-3.7
S4B(Mo)	96	3.6	-3.1	3.5	-3.4	3.5	-3.3	3.5	-3.7	93	3.6	-3.2	3.5	-3.4	3.6	-3.4	3.5	-3.7
S4B(Fe1)	95	3.6	-3.2	3.5	-3.4	3.5	-3.3	3.5	-3.7	81	3.6	-3.2	3.6	-3.4	3.5	-3.4	3.5	-3.7
S2B(3)	0	3.6	-3.1	3.5	-3.4	3.6	-3.4	3.6	-3.8	0	3.6	-3.2	3.5	-3.5	3.6	-3.4	3.6	-3.8
S2B(5)	7	3.6	-3.2	3.5	-3.4	3.6	-3.3	3.6	-3.8	6	3.6	-3.3	3.5	-3.5	3.6	-3.4	3.6	-3.8
S3A(2)	57	3.6	-3.4	3.5	-3.1	3.5	-3.4	3.5	-3.7	54	3.6	-3.5	3.6	-3.3	3.6	-3.5	3.6	-3.7
S3A(5)	37	3.6	-3.4	3.5	-3.1	3.5	-3.4	3.5	-3.7	33	3.6	-3.5	3.6	-3.2	3.6	-3.5	3.6	-3.7
S5A(2)	73	3.6	-3.1	3.4	-3.4	3.6	-3.4	3.5	-3.7	70	3.6	-3.2	3.4	-3.4	3.6	-3.4	3.5	-3.7
S5A(3)	85	3.6	-3.4	3.4	-3.3	3.6	-3.2	3.5	-3.7	80	3.6	-3.4	3.5	-3.3	3.6	-3.4	3.5	-3.7
Fe1	116	2.9	-3.1	3.5	-3.2	3.5	-2.8	3.6	-3.8	120	2.9	-3.2	3.6	-3.2	3.6	-2.9	3.6	-3.7
Fe2	22	3.6	-2.9	3.4	-3.5	3.5	-3.4	3.6	-3.8	36	3.6	-2.9	3.5	-3.5	3.6	-3.4	3.6	-3.7
Fe3	97	3.6	-3.3	3.2	-3.2	3.7	-3.1	3.6	-3.7	111	3.6	-3.4	3.1	-3.3	3.7	-3.2	3.6	-3.7
Fe4	52	3.6	-3.4	3.4	-3.0	3.6	-3.4	3.5	-3.8	67	3.6	-3.4	3.5	-3.0	3.6	-3.5	3.6	-3.7
Fe5	91	3.6	-3.3	3.5	-3.2	3.5	-3.3	3.6	-3.8	109	3.6	-3.4	3.6	-3.1	3.2	-3.3	3.6	-3.7
Fe6	41	3.6	-3.4	3.5	-3.4	3.5	-3.0	3.6	-3.8	55	3.6	-3.4	3.5	-3.5	3.6	-3.1	3.6	-3.7
Fe7	79	3.5	-2.8	3.4	-3.3	3.5	-3.0	3.0	-3.8	85	3.6	-2.8	3.5	-3.3	3.6	-3.1	3.0	-3.7
Fe2/6(3)	76	3.6	-3.4	3.5	-3.5	3.5	-3.1	3.6	-3.8	87	3.6	-3.4	3.5	-3.5	3.6	-3.2	3.6	-3.8
Fe2/6(5)	63	3.6	-3.3	3.5	-3.5	3.5	-3.1	3.5	-3.8	75	3.6	-3.5	3.5	-3.5	3.6	-3.1	3.6	-3.7
Fe3/7(2)	91	3.6	-2.9	3.1	-3.3	3.7	-3.2	3.4	-3.8	96	3.6	-3.0	3.2	-3.4	3.7	-3.3	3.4	-3.7
Fe3/7(3)	91	3.6	-2.8	3.0	-3.3	3.6	-3.2	3.5	-3.8	88	3.7	-2.8	3.1	-3.4	3.6	-3.3	3.5	-3.7
Fe4/5(2)	96	3.6	-3.5	3.6	-3.0	3.6	-3.6	3.6	-3.8	63	3.6	-3.5	3.5	-3.0	3.7	-3.7	3.6	-3.8
Fe4/5(5)	75	3.6	-3.5	3.4	-2.9	3.5	-3.5	3.5	-3.8	90	3.6	-3.5	3.5	-3.0	3.6	-3.5	3.6	-3.7
Fe5/8	134	3.6	-3.4	3.5	-3.5	3.4	-3.4	3.6	-3.3	150	3.6	-3.5	3.5	-3.5	3.5	-3.5	3.6	-3.1
Fe7/8	134	3.6	-3.4	3.5	-3.5	3.5	-3.4	3.5	-3.4	149	3.6	-3.5	3.5	-3.5	3.6	-3.5	3.5	-3.2

Table S19. Relative energies (ΔE in kJ/mol) and spin densities of the various BS states for the E_1 state of Fe-nitrogenase, calculated with r^{2}SCAN and TPSSh with the large QM system using the S2B(3) protonation state.

BS	r^{2}SCAN									TPSSh								
	ΔE	Fe1	Fe2	Fe3	Fe4	Fe5	Fe6	Fe7	Fe8	ΔE	Fe1	Fe2	Fe3	Fe4	Fe5	Fe6	Fe7	Fe8
1234	85	-3.7	-3.2	-3.4	-3.4	3.4	3.5	3.6	3.9	89	-3.7	-3.3	-3.4	-3.4	3.5	3.5	3.6	3.8
1235	20	-3.5	-3.3	-3.4	3.6	-3.5	3.4	3.5	3.8	40	-3.6	-3.5	-3.5	3.6	-3.3	3.5	3.6	3.8
1236	26	-3.6	-3.3	-3.5	3.5	3.5	-3.5	3.6	3.8	35	-3.6	-3.3	-3.5	3.6	3.5	-3.5	3.6	3.8
1237	25	-3.6	-3.4	-3.4	3.6	3.5	3.6	-3.5	3.8	37	-3.6	-3.4	-3.4	3.6	3.5	3.6	-3.5	3.8
1238	51	-3.6	-3.3	-3.4	3.6	3.6	3.5	3.6	-3.6	48	-3.6	-3.4	-3.4	3.6	3.7	3.6	3.6	-3.7
1245	19	-3.5	-3.4	3.5	-3.4	-3.5	3.5	3.5	3.8	32	-3.6	-3.4	3.6	-3.4	-3.5	3.6	3.5	3.8
1246	12	-3.5	-3.4	3.5	-3.5	3.6	-3.5	3.5	3.8	22	-3.6	-3.4	3.5	-3.5	3.6	-3.5	3.6	3.8
1247	10	-3.5	-3.3	3.6	-3.4	3.5	3.5	-3.5	3.8	29	-3.6	-3.5	3.6	-3.5	3.5	3.5	-3.3	3.8
1248	36	-3.5	-3.3	3.6	-3.4	3.6	3.5	3.6	-3.7	36	-3.6	-3.4	3.6	-3.4	3.6	3.6	3.7	-3.7
1256	8	-3.6	-3.3	3.5	3.6	-3.5	-3.2	3.4	3.8	0	-3.6	-3.4	3.6	3.6	-3.5	-3.3	3.5	3.8
1257	22	-3.6	-3.2	3.5	3.5	-3.4	3.6	-3.4	3.8	9	-3.6	-3.3	3.5	3.5	-3.4	3.6	-3.5	3.8
1258	28	-3.6	-3.1	3.6	3.6	-3.2	3.5	3.6	-3.8	30	-3.4	-3.4	3.6	3.6	-3.5	3.5	3.6	-3.8
1267	10	-3.6	-3.3	3.5	3.5	3.5	-3.2	-3.5	3.8	0	-3.6	-3.4	3.6	3.6	3.5	-3.3	-3.5	3.8
1268	13	-3.6	-3.2	3.6	3.6	3.6	-3.2	3.6	-3.8	4	-3.6	-3.3	3.6	3.6	3.6	-3.3	3.6	-3.8
1278	36	-3.6	-3.2	3.5	3.6	3.6	3.5	-3.2	-3.7	36	-3.6	-3.3	3.5	3.6	3.6	3.6	-3.3	-3.7
1345	24	-3.5	3.5	-3.4	-3.3	-3.5	3.4	3.6	3.8	38	-3.5	3.5	-3.4	-3.4	-3.5	3.5	3.6	3.8
1346	38	-3.5	3.5	-3.5	-3.5	3.5	-3.2	3.5	3.8	53	-3.7	3.3	-3.4	-3.5	3.6	-3.2	3.7	3.8
1347	19	-3.5	3.5	-3.3	-3.4	3.6	3.5	-3.5	3.8	32	-3.5	3.5	-3.4	-3.4	3.6	3.5	-3.5	3.8
1348	38	-3.5	3.5	-3.3	-3.3	3.6	3.5	3.6	-3.6	39	-3.6	3.6	-3.4	-3.4	3.6	3.5	3.6	-3.7
1356	18	-3.6	3.4	-3.4	3.4	-3.4	-3.0	3.6	3.8	17	-3.6	3.5	-3.4	3.5	-3.5	-3.4	3.6	3.8
1357	26	-3.6	3.5	-3.4	3.6	-3.4	3.4	-3.2	3.8	26	-3.6	3.5	-3.4	3.6	-3.4	3.5	-3.5	3.8
1358	33	-3.5	3.5	-3.2	3.5	-3.2	3.6	3.6	-3.8	29	-3.5	3.5	-3.3	3.6	-3.3	3.6	3.6	-3.8
1367	19	-3.5	3.5	-3.4	3.5	3.5	-3.4	-3.2	3.8	18	-3.6	3.5	-3.4	3.5	3.5	-3.4	-3.3	3.8
1368	42	-3.5	3.5	-3.3	3.5	3.6	-3.1	3.6	-3.8	38	-3.5	3.5	-3.3	3.6	3.6	-3.3	3.6	-3.8
1378	34	-3.5	3.5	-3.2	3.5	3.6	3.6	-3.2	-3.7	21	-3.6	3.6	-3.3	3.6	3.6	3.6	-3.3	-3.7
1456	29	-3.6	3.5	3.5	-3.3	-3.3	-3.3	3.5	3.8	28	-3.6	3.6	3.5	-3.4	-3.2	-3.4	3.5	3.8
1457	28	-3.6	3.5	3.6	-3.3	-3.2	3.4	-3.4	3.8	19	-3.6	3.5	3.6	-3.4	-3.2	3.5	-3.4	3.8
1458	26	-3.6	3.6	3.6	-3.2	-3.1	3.6	3.6	-3.8	20	-3.6	3.6	3.6	-3.3	-3.3	3.6	3.6	-3.8
1467	12	-3.6	3.4	3.4	-3.5	3.5	-3.0	-3.4	3.8	13	-3.6	3.4	3.5	-3.5	3.6	-3.1	-3.4	3.8
1468	31	-3.6	3.5	3.5	-3.3	3.6	-3.1	3.6	-3.8	26	-3.6	3.5	3.6	-3.4	3.6	-3.2	3.6	-3.7
1478	33	-3.5	3.5	3.5	-3.2	3.5	3.6	-3.1	-3.7	29	-3.6	3.5	3.5	-3.3	3.6	3.6	-3.3	-3.7
1567	52	-3.3	3.5	3.6	3.5	-3.4	-3.2	-3.4	3.7	29	-3.4	3.5	3.6	3.6	-3.5	-3.3	-3.4	3.7
1568	26	-3.3	3.5	3.6	3.6	-3.3	-3.3	3.6	-3.8	12	-3.4	3.6	3.7	3.6	-3.3	-3.4	3.6	-3.8
1578	39	-3.3	3.5	3.6	3.6	-3.2	3.6	-3.3	-3.8	25	-3.4	3.5	3.6	3.6	-3.3	3.6	-3.3	-3.8
1678	34	-3.3	3.5	3.6	3.6	3.6	-3.2	-3.4	-3.8	36	-3.4	3.6	3.6	3.5	3.7	-3.3	-3.4	-3.8
2345	27	3.7	-3.3	-3.4	-3.4	-3.6	3.3	3.4	3.8	41	3.4	-3.3	-3.3	-3.4	-3.6	3.5	3.6	3.8
2346	26	3.4	-3.4	-3.4	-3.3	3.5	-3.5	3.5	3.8	27	3.4	-3.4	-3.4	-3.4	3.6	-3.5	3.6	3.8
2347	31	3.4	-3.3	-3.4	-3.3	3.5	3.5	-3.5	3.8	32	3.4	-3.3	-3.4	-3.3	3.6	3.5	-3.6	3.8
2348	57	3.2	-3.1	-3.3	-3.3	3.5	3.5	3.5	-3.7	43	3.3	-3.2	-3.4	-3.4	3.6	3.5	3.6	-3.7

2356	3	3.6	-3.3	-3.4	3.5	-3.5	-3.4	3.4	3.8	12	3.6	-3.3	-3.5	3.5	-3.5	-3.5	3.5	3.8
2357	18	3.6	-3.4	-3.3	3.5	-3.5	3.4	-3.4	3.8	25	3.6	-3.4	-3.4	3.5	-3.5	3.5	-3.5	3.8
2358	10	3.6	-3.0	-3.3	3.5	-3.5	3.5	3.5	-3.8	3	3.6	-3.4	-3.4	3.6	-3.3	3.5	3.5	-3.7
2367	10	3.6	-3.4	-3.4	3.4	3.4	-3.4	-3.4	3.8	21	3.6	-3.4	-3.4	3.5	3.5	-3.4	-3.5	3.8
2368	15	3.6	-3.2	-3.4	3.5	3.6	-3.4	3.6	-3.8	15	3.6	-3.3	-3.5	3.5	3.6	-3.4	3.6	-3.8
2378	20	3.6	-3.4	-3.2	3.5	3.5	3.5	-3.4	-3.8	19	3.6	-3.4	-3.3	3.5	3.6	3.6	-3.4	-3.7
2456	23	3.6	-3.3	3.5	-3.4	-3.5	-3.4	3.4	3.8	35	3.6	-3.3	3.5	-3.4	-3.5	-3.4	3.5	3.8
2457	27	3.6	-3.4	3.5	-3.3	-3.4	3.4	-3.5	3.8	34	3.6	-3.4	3.6	-3.3	-3.5	3.4	-3.5	3.8
2458	22	3.6	-3.1	3.5	-3.4	-3.3	3.5	3.5	-3.8	20	3.6	-3.4	3.5	-3.3	-3.4	3.6	3.6	-3.8
2467	0	3.6	-3.3	3.5	-3.4	3.4	-3.4	-3.5	3.8	5	3.6	-3.3	3.5	-3.5	3.5	-3.5	-3.5	3.8
2468	10	3.6	-3.1	3.5	-3.4	3.6	-3.4	3.6	-3.8	10	3.6	-3.2	3.5	-3.5	3.6	-3.4	3.6	-3.8
2478	17	3.6	-3.1	3.6	-3.4	3.5	3.4	-3.3	-3.8	5	3.6	-3.4	3.6	-3.4	3.5	3.5	-3.3	-3.7
2567	35	3.5	-3.5	3.4	3.4	-3.3	-3.4	-3.4	3.9	43	3.5	-3.5	3.5	3.5	-3.3	-3.4	-3.3	3.7
2568	33	3.6	-3.4	3.5	3.6	-3.4	-3.2	3.6	-3.8	28	3.7	-3.4	3.6	3.6	-3.4	-3.3	3.6	-3.8
2578	56	3.7	-3.1	3.5	3.5	-3.4	3.5	-3.5	-3.8	48	3.6	-3.2	3.6	3.6	-3.4	3.6	-3.5	-3.8
2678	51	3.7	-3.2	3.5	3.6	3.5	-3.3	-3.5	-3.8	41	3.7	-3.4	3.6	3.6	3.6	-3.3	-3.5	-3.8
3456	20	3.6	3.4	-3.4	-3.3	-3.4	-3.4	3.5	3.8	30	3.6	3.5	-3.4	-3.4	-3.4	-3.5	3.5	3.8
3457	26	3.6	3.4	-3.4	-3.3	-3.4	3.4	-3.4	3.8	40	3.6	3.5	-3.4	-3.4	-3.5	3.5	-3.5	3.8
3458	16	3.6	3.4	-3.4	-3.1	-3.3	3.5	3.6	-3.8	16	3.6	3.5	-3.4	-3.2	-3.4	3.6	3.6	-3.8
3467	7	3.6	3.4	-3.4	-3.4	3.5	-3.5	-3.4	3.8	13	3.6	3.5	-3.5	-3.4	3.5	-3.5	-3.4	3.8
3468	11	3.6	3.5	-3.4	-3.3	3.5	-3.2	3.5	-3.7	3	3.7	3.5	-3.4	-3.4	3.5	-3.3	3.5	-3.7
3478	14	3.6	3.4	-3.2	-3.4	3.6	3.5	-3.4	-3.8	14	3.6	3.5	-3.2	-3.4	3.6	3.6	-3.4	-3.7
3567	21	3.5	3.4	-3.5	3.4	-3.3	-3.3	-3.3	3.9	37	3.6	3.5	-3.4	3.5	-3.3	-3.3	-3.4	3.7
3568	40	3.7	3.5	-3.4	3.5	-3.4	-3.2	3.7	-3.8	42	3.6	3.5	-3.3	3.6	-3.5	-3.4	3.7	-3.8
3578	36	3.7	3.4	-3.4	3.6	-3.3	3.6	-3.3	-3.7	36	3.7	3.5	-3.4	3.6	-3.4	3.6	-3.2	-3.8
3678	40	3.7	3.6	-3.5	3.5	3.6	-3.3	-3.3	-3.8	37	3.7	3.6	-3.4	3.6	3.6	-3.4	-3.3	-3.8
4567	31	3.6	3.4	3.5	-3.5	-3.3	-3.3	-3.4	3.9	46	3.6	3.4	3.5	-3.5	-3.3	-3.3	-3.4	3.8
4568	55	3.7	3.6	3.5	-3.5	-3.3	-3.3	3.6	-3.7	58	3.7	3.6	3.6	-3.4	-3.3	-3.4	3.6	-3.8
4578	56	3.7	3.4	3.6	-3.5	-3.3	3.5	-3.3	-3.7	58	3.7	3.6	3.6	-3.4	-3.2	3.5	-3.5	-3.8
4678	50	3.6	3.6	3.5	-3.2	3.6	-3.4	-3.4	-3.8	43	3.6	3.6	3.6	-3.3	3.7	-3.4	-3.5	-3.8
5678	100	3.7	3.5	3.6	3.7	-3.4	-3.2	-3.5	-3.9	103	3.7	3.6	3.6	3.7	-3.4	-3.3	-3.5	-3.9

Table S20. Relative energies (Δ*E* in kJ/mol) and spin densities of the various BS states for the E_1 state of Fe-nitrogenase, calculated with B3LYP and TPSS with the large QM system using the S2B(3) protonation state.

BS	B3LYP									TPSS								
	Δ*E*	Fe1	Fe2	Fe3	Fe4	Fe5	Fe6	Fe7	Fe8	Δ*E*	Fe1	Fe2	Fe3	Fe4	Fe5	Fe6	Fe7	Fe8
1234	157	-3.7	-3.5	-3.6	-3.6	3.7	3.7	3.8	3.8	129	-3.6	-2.7	-2.9	-3.0	3.0	3.1	3.1	3.8
1235	142	-3.6	-3.5	-3.5	3.8	-3.7	3.6	3.7	3.8	43	-3.3	-2.8	-2.9	3.1	-3.1	3.0	3.1	3.7
1236	127	-3.7	-3.5	-3.6	3.7	3.7	-3.6	3.7	3.8	69	-3.3	-3.1	-3.0	3.0	3.1	-3.0	3.2	3.7
1237	120	-3.7	-3.5	-3.6	3.7	3.6	3.7	-3.6	3.8	66	-3.4	-2.9	-3.1	3.1	3.1	3.2	-3.0	3.7
1238	105	-3.7	-3.5	-3.6	3.8	3.7	3.7	3.7	-3.7	63	-3.1	-2.9	-2.1	3.0	3.1	3.0	3.1	-3.4
1245	133	-3.6	-3.5	3.7	-3.5	-3.7	3.7	3.6	3.8	51	-3.3	-3.0	3.0	-3.0	-3.0	3.2	3.1	3.7
1246	64	-3.7	-3.5	3.7	-3.6	3.8	-3.6	3.7	3.8	57	-3.3	-3.1	3.0	-3.1	3.2	-2.9	3.2	3.7
1247	132	-3.6	-3.5	3.8	-3.5	3.7	3.6	-3.7	3.8	37	-3.3	-2.9	3.1	-3.0	3.1	3.0	-3.1	3.7
1248	77	-3.6	-3.5	3.7	-3.6	3.7	3.7	3.7	-3.7	62	-3.3	-2.7	3.0	-2.4	3.2	3.1	3.1	-3.4
1256	86	-3.7	-3.6	3.7	3.7	-3.6	-3.5	3.7	3.7	18	-3.3	-3.0	3.0	3.1	-3.1	-2.5	2.9	3.6
1257	88	-3.6	-3.5	3.6	3.7	-3.6	3.8	-3.6	3.8	12	-3.3	-2.4	2.9	3.0	-3.0	3.0	-3.0	3.6
1258	100	-3.7	-3.5	3.7	3.8	-3.6	3.7	3.7	-3.8	34	-3.4	-2.4	3.3	3.1	-2.7	3.1	3.3	-3.6
1267	88	-3.7	-3.6	3.7	3.7	3.7	-3.5	-3.6	3.8	18	-3.3	-3.0	3.0	3.0	2.9	-2.5	-3.1	3.6
1268	49	-3.7	-3.5	3.7	3.7	3.7	-3.5	3.7	-3.8	39	-3.4	-2.7	3.2	3.2	3.3	-2.6	3.3	-3.6
1278	107	-3.7	-3.5	3.7	3.7	3.7	3.7	-3.5	-3.7	43	-3.3	-2.5	3.0	3.2	3.3	3.1	-2.6	-3.6
1345	125	-3.6	3.7	-3.5	-3.5	-3.6	3.6	3.8	3.8	57	-3.2	2.9	-3.0	-3.0	-3.0	3.1	3.2	3.7
1346	141	-3.6	3.7	-3.6	-3.6	3.7	-3.5	3.7	3.8	59	-3.2	2.8	-3.1	-3.1	3.1	-2.5	3.1	3.7
1347	127	-3.6	3.7	-3.5	-3.5	3.8	3.6	-3.6	3.8	56	-3.3	2.9	-2.9	-3.0	3.2	3.1	-3.0	3.7
1348	108	-3.6	3.7	-3.5	-3.5	3.7	3.6	3.7	-3.7	65	-3.1	3.0	-2.8	-2.8	3.2	3.2	3.2	-3.3
1356	109	-3.6	3.6	-3.6	3.7	-3.6	-3.5	3.8	3.8	5	-3.2	2.7	-3.1	2.9	-2.8	-2.3	3.1	3.6
1357	99	-3.6	3.6	-3.6	3.8	-3.6	3.6	-3.6	3.8	31	-3.2	3.0	-2.9	3.1	-3.0	2.9	-2.5	3.6
1358	94	-3.6	3.6	-3.6	3.8	-3.5	3.6	3.7	-3.8	42	-3.2	3.2	-2.6	3.1	-2.6	3.3	3.2	-3.6
1367	100	-3.6	3.7	-3.6	3.7	3.7	-3.6	-3.5	3.8	27	-3.2	3.0	-3.0	3.1	3.0	-3.0	-2.6	3.6
1368	68	-3.6	3.6	-3.6	3.7	3.7	-3.5	3.8	-3.8	44	-3.2	3.0	-2.8	3.2	3.3	-2.3	3.1	-3.6
1378	115	-3.6	3.6	-3.5	3.7	3.7	3.7	-3.5	-3.7	50	-3.2	3.1	-2.7	3.2	3.3	3.2	-2.6	-3.6
1456	75	-3.6	3.7	3.7	-3.6	-3.6	-3.5	3.7	3.8	33	-3.3	3.0	3.1	-3.0	-2.6	-2.8	3.0	3.6
1457	106	-3.6	3.6	3.8	-3.6	-3.5	3.6	-3.6	3.8	29	-3.4	3.1	3.2	-2.9	-2.5	2.9	-3.0	3.6
1458	50	-3.7	3.7	3.7	-3.5	-3.5	3.7	3.7	-3.8	45	-3.4	3.2	3.2	-2.6	-2.4	3.2	3.3	-3.6
1467	103	-3.7	3.6	3.7	-3.7	3.7	-3.4	-3.5	3.8	4	-3.3	2.8	2.9	-3.1	3.1	-2.3	-2.8	3.6
1468	55	-3.6	3.6	3.7	-3.6	3.8	-3.5	3.7	-3.8	39	-3.3	3.0	3.2	-2.8	3.1	-2.3	3.3	-3.6
1478	76	-3.6	3.6	3.7	-3.5	3.7	3.6	-3.5	-3.7	42	-3.3	3.2	3.1	-2.6	3.2	3.3	-2.5	-3.6
1567	115	-3.5	3.6	3.8	3.7	-3.6	-3.5	-3.6	3.7	35	-3.0	3.1	2.8	3.2	-2.8	-2.7	-2.8	3.3
1568	93	-3.6	3.6	3.7	3.7	-3.5	-3.5	3.8	-3.8	42	-3.0	3.1	3.3	3.3	-2.5	-2.6	3.2	-3.7
1578	111	-3.6	3.6	3.7	3.7	-3.5	3.7	-3.5	-3.8	49	-2.9	3.2	3.2	3.2	-2.8	3.2	-2.8	-3.5
1678	82	-3.6	3.6	3.7	3.7	3.8	-3.5	-3.6	-3.8	49	-3.0	3.1	3.1	3.2	3.2	-2.3	-2.7	-3.7
2345	135	3.8	-3.5	-3.6	-3.5	-3.7	3.5	3.5	3.8	40	3.2	-2.9	-2.9	-3.0	-3.2	2.9	3.0	3.6
2346	64	3.6	-3.5	-3.6	-3.6	3.7	-3.6	3.7	3.8	37	3.1	-3.1	-3.0	-3.0	3.1	-3.1	3.0	3.6
2347	75	3.6	-3.5	-3.5	-3.5	3.7	3.7	-3.7	3.8	35	3.0	-2.9	-3.0	-2.9	3.0	2.9	-3.1	3.6
2348	75	3.5	-3.5	-3.6	-3.6	3.8	3.7	3.7	-3.7	37	2.7	-2.3	-2.8	-2.7	3.0	3.1	3.0	-3.3

2356	99	3.7	-3.6	-3.6	3.7	-3.6	-3.6	3.7	3.7	14	3.3	-3.0	-3.0	3.1	-3.1	-3.1	2.9	3.6
2357	89	3.7	-3.6	-3.6	3.7	-3.6	3.7	-3.6	3.8	20	3.3	-2.9	-2.9	3.1	-3.1	2.7	-3.1	3.6
2358	69	3.7	-3.5	-3.6	3.7	-3.6	3.6	3.7	-3.8	3	3.3	-2.3	-2.6	3.2	-3.1	2.9	3.0	-3.6
2367	91	3.7	-3.6	-3.6	3.7	3.7	-3.5	-3.6	3.8	24	3.3	-3.0	-3.0	3.0	2.9	-3.0	-3.1	3.6
2368	0	3.7	-3.6	-3.6	3.7	3.7	-3.6	3.8	-3.8	26	3.3	-2.4	-3.0	3.0	3.2	-2.9	3.2	-3.6
2378	79	3.7	-3.6	-3.6	3.7	3.7	3.7	-3.6	-3.7	32	3.2	-2.8	-2.5	3.1	3.2	3.1	-2.9	-3.6
2456	111	3.7	-3.5	3.7	-3.6	-3.6	-3.6	3.7	3.7	31	3.2	-2.9	3.0	-2.9	-3.1	-3.0	2.9	3.6
2457	23	3.7	-3.6	3.8	-3.6	-3.7	3.7	-3.6	3.8	22	3.2	-2.8	3.1	-2.7	-3.1	2.6	-3.1	3.6
2458	51	3.7	-3.5	3.7	-3.6	-3.6	3.7	3.7	-3.8	29	3.2	-2.5	3.1	-2.7	-2.9	3.1	3.2	-3.6
2467	93	3.7	-3.5	3.7	-3.6	3.7	-3.6	-3.6	3.8	11	3.3	-2.8	3.1	-3.0	2.9	-3.1	-3.2	3.6
2468	8	3.7	-3.6	3.7	-3.6	3.8	-3.6	3.7	-3.8	20	3.2	-2.3	3.0	-3.0	3.2	-2.9	3.2	-3.6
2478	71	3.7	-3.5	3.7	-3.6	3.6	3.6	-3.5	-3.7	7	3.2	-2.4	3.2	-2.8	3.0	2.9	-2.9	-3.6
2567	187	3.6	-3.7	3.5	3.5	-3.6	-3.4	-3.5	4.0	46	3.2	-3.0	3.0	3.0	-2.9	-3.1	-3.0	3.6
2568	93	3.7	-3.6	3.7	3.8	-3.6	-3.5	3.7	-3.8	61	3.3	-2.8	3.1	3.2	-2.8	-2.3	3.0	-3.7
2578	126	3.7	-3.5	3.7	3.7	-3.6	3.7	-3.6	-3.8	78	3.4	-2.3	3.1	3.2	-3.0	3.0	-3.0	-3.7
2678	101	3.7	-3.6	3.7	3.7	3.7	-3.5	-3.6	-3.8	80	3.5	-2.5	3.2	3.3	3.0	-3.0	-3.2	-3.7
3456	37	3.7	3.6	-3.6	-3.7	-3.7	-3.5	3.8	3.8	23	3.3	2.9	-2.9	-2.8	-3.1	-3.1	2.9	3.6
3457	53	3.7	3.7	-3.6	-3.5	-3.6	3.7	-3.7	3.8	33	3.3	3.0	-2.9	-2.9	-3.0	2.9	-3.1	3.6
3458	100	3.7	3.6	-3.5	-3.5	-3.6	3.6	3.7	-3.8	25	3.3	3.0	-2.8	-2.5	-2.8	3.2	3.2	-3.6
3467	81	3.7	3.6	-3.7	-3.6	3.7	-3.6	-3.6	3.8	14	3.3	2.9	-2.9	-3.0	3.0	-3.0	-3.0	3.6
3468	92	3.7	3.7	-3.6	-3.6	3.7	-3.5	3.7	-3.7	0	3.3	3.0	-2.8	-2.9	3.0	-2.4	3.0	-3.5
3478	79	3.7	3.6	-3.5	-3.6	3.8	3.7	-3.6	-3.7	24	3.3	3.0	-2.4	-2.9	3.2	3.2	-2.8	-3.6
3567	153	3.6	3.6	-3.6	3.6	-3.5	-3.4	-3.5	3.7	38	3.3	2.9	-3.0	3.0	-2.9	-3.0	-3.1	3.6
3568	114	3.7	3.6	-3.5	3.7	-3.6	-3.6	3.8	-3.8	44	3.5	3.0	-3.2	3.1	-2.6	-2.4	3.1	-3.6
3578	121	3.7	3.6	-3.6	3.8	-3.5	3.7	-3.5	-3.8	58	3.5	3.2	-3.0	3.2	-2.8	3.1	-2.6	-3.6
3678	122	3.7	3.7	-3.6	3.7	3.7	-3.5	-3.5	-3.8	66	3.5	3.2	-3.1	3.1	3.1	-2.7	-2.8	-3.7
4567	149	3.7	3.6	3.7	-3.6	-3.5	-3.4	-3.5	3.7	50	3.4	2.9	3.0	-3.1	-3.0	-3.0	-3.0	3.6
4568	75	3.7	3.7	3.7	-3.6	-3.6	-3.5	3.7	-3.8	71	3.3	3.0	2.8	-2.9	-1.9	-2.7	3.1	-3.6
4578	141	3.7	3.6	3.8	-3.6	-3.5	3.7	-3.6	-3.8	74	3.4	3.1	3.1	-3.0	-2.5	3.0	-2.7	-3.6
4678	131	3.7	3.6	3.7	-3.5	3.8	-3.6	-3.6	-3.8	56	3.4	3.1	3.2	-3.0	3.2	-2.7	-2.9	-3.6
5678	113	3.7	3.6	3.7	3.8	-3.6	-3.5	-3.6	-3.8	136	3.7	3.1	3.1	3.3	-2.7	-2.9	-3.0	-3.8

Table S21. Relative energies (Δ*E* in kJ/mol) and spin densities of the various BS states for the E_1 state of Fe-nitrogenase, calculated with r^2SCAN and TPSSh with the large QM system using the Fe2 protonation state.

BS	r^2SCAN									TPSSh								
	Δ*E*	Fe1	Fe2	Fe3	Fe4	Fe5	Fe6	Fe7	Fe8	Δ*E*	Fe1	Fe2	Fe3	Fe4	Fe5	Fe6	Fe7	Fe8
1234	117	-3.8	-3.1	-3.3	-3.4	3.5	3.4	3.5	3.9	122	-3.8	-3.0	-3.4	-3.4	3.5	3.5	3.5	3.9
1236	55	-3.6	-3.1	-3.5	3.5	3.5	-3.5	3.6	3.8	64	-3.7	-3.1	-3.5	3.5	3.5	-3.5	3.6	3.8
1237	71	-3.6	-3.2	-3.4	3.5	3.4	3.5	-3.5	3.8	71	-3.7	-3.2	-3.5	3.6	3.5	3.6	-3.5	3.8
1245	62	-3.5	-3.1	3.5	-3.4	-3.5	3.5	3.4	3.8	64	-3.7	-3.1	3.5	-3.5	-3.5	3.6	3.5	3.8
1246	41	-3.7	-3.1	3.5	-3.5	3.6	-3.4	3.6	3.8	40	-3.7	-3.1	3.5	-3.5	3.6	-3.5	3.6	3.8
1247	48	-3.5	-3.1	3.5	-3.5	3.4	3.4	-3.6	3.8	61	-3.6	-3.1	3.6	-3.5	3.5	3.5	-3.6	3.8
1256	21	-3.6	-3.0	3.5	3.5	-3.5	-3.5	3.4	3.8	23	-3.6	-3.1	3.5	3.6	-3.5	-3.5	3.5	3.8
1257	13	-3.6	-3.1	3.4	3.5	-3.4	3.5	-3.4	3.8	11	-3.7	-3.2	3.5	3.5	-3.4	3.6	-3.4	3.7
1258	32	-3.6	-3.1	3.5	3.5	-3.3	3.6	3.6	-3.8	41	-3.6	-3.1	3.6	3.6	-3.4	3.6	3.6	-3.8
1267	21	-3.6	-3.0	3.5	3.5	3.5	-3.5	-3.5	3.8	30	-3.6	-3.1	3.5	3.5	3.5	-3.5	-3.5	3.8
1278	40	-3.6	-3.1	3.5	3.5	3.6	3.6	-3.3	-3.8	48	-3.6	-3.1	3.5	3.6	3.6	3.6	-3.4	-3.8
1345	70	-3.5	3.0	-3.3	-3.2	-3.5	3.5	3.6	3.8	87	-3.5	3.0	-3.3	-3.1	-3.4	3.6	3.6	3.8
1346	56	-3.5	3.2	-3.2	-3.3	3.6	-3.5	3.6	3.8	76	-3.7	3.1	-3.4	-3.5	3.6	-3.1	3.7	3.8
1348	99	-3.5	3.1	-3.3	-3.3	3.6	3.7	3.6	-3.7									
1356	43	-3.6	3.1	-3.3	3.5	-3.3	-3.1	3.5	3.8	44	-3.5	3.0	-3.2	3.5	-3.4	-3.2	3.6	3.8
1357	53	-3.6	2.9	-3.2	3.5	-3.2	3.5	-3.1	3.8	55	-3.6	2.9	-3.2	3.5	-3.3	3.5	-3.0	3.7
1358	69	-3.5	3.1	-3.2	3.6	-3.0	3.6	3.6	-3.8	64	-3.6	3.1	-3.3	3.7	-3.1	3.6	3.6	-3.8
1367	56	-3.5	3.1	-3.3	3.5	3.5	-3.2	-3.1	3.8	80	-3.6	3.1	-3.3	3.5	3.6	-3.1	-3.5	3.8
1368	72	-3.6	3.3	-3.2	3.6	3.6	-3.1	3.6	-3.8	73	-3.5	3.1	-3.2	3.6	3.6	-3.1	3.6	-3.7
1378	52	-3.5	3.1	-3.0	3.6	3.6	3.6	-3.2	-3.8	60	-3.5	3.1	-3.1	3.6	3.6	3.6	-3.3	-3.7
1456	70	-3.6	3.1	3.5	-3.3	-3.1	-3.2	3.5	3.8	79	-3.6	3.0	3.6	-3.4	-3.1	-3.2	3.5	3.8
1457	56	-3.6	2.9	3.5	-3.2	-2.8	3.5	-3.4	3.8	51	-3.6	3.0	3.5	-3.2	-2.9	3.5	-3.4	3.7
1458	55	-3.6	3.1	3.6	-3.0	-3.1	3.6	3.6	-3.8	64	-3.6	3.1	3.6	-3.1	-3.2	3.6	3.6	-3.8
1467	37	-3.6	3.1	3.4	-3.4	3.5	-3.0	-3.3	3.8	47	-3.6	3.0	3.5	-3.5	3.6	-3.0	-3.2	3.8
1468	63	-3.5	3.2	3.6	-3.2	3.6	-3.0	3.6	-3.8	65	-3.6	3.2	3.6	-3.3	3.6	-3.1	3.6	-3.7
1478	69	-3.5	3.0	3.6	-3.2	3.7	3.7	-3.1	-3.8	74	-3.6	3.0	3.6	-3.3	3.7	3.7	-3.2	-3.7
1567	57	-3.7	2.8	3.5	3.5	-3.1	-3.0	-3.2	3.9	70	-3.4	3.1	3.6	3.6	-3.4	-2.8	-3.5	3.7
1568	55	-3.3	3.2	3.6	3.6	-3.2	-3.1	3.6	-3.8	47	-3.4	3.1	3.6	3.6	-3.3	-3.1	3.6	-3.8
1578	59	-3.3	3.1	3.7	3.7	-3.2	3.6	-3.3	-3.8	51	-3.4	3.1	3.7	3.7	-3.3	3.7	-3.3	-3.8
1678	60	-3.3	3.2	3.6	3.6	3.6	-3.1	-3.2	-3.8	52	-3.4	3.2	3.6	3.6	3.6	-3.1	-3.3	-3.8
2345	41	3.7	-2.8	-3.4	-3.4	-3.6	3.1	3.3	3.8	57	3.4	-3.0	-3.4	-3.6	-3.6	3.5	3.6	3.8
2346	44	3.3	-3.0	-3.5	-3.5	3.5	-3.5	3.6	3.8	44	3.4	-3.1	-3.5	-3.5	3.6	-3.6	3.6	3.8
2347	48	3.4	-3.0	-3.4	-3.5	3.5	3.5	-3.6	3.8	42	3.4	-3.0	-3.5	-3.5	3.5	3.5	-3.6	3.8
2348	66	3.6	-2.7	-3.3	-3.3	3.4	3.4	3.3	-3.8	61	3.4	-2.8	-3.4	-3.4	3.5	3.5	3.5	-3.7
2356	6	3.6	-3.0	-3.5	3.4	-3.5	-3.5	3.4	3.8	17	3.6	-3.0	-3.5	3.5	-3.5	-3.6	3.5	3.8
2357	19	3.6	-3.0	-3.5	3.5	-3.5	3.4	-3.6	3.8	27	3.6	-3.0	-3.5	3.5	-3.5	3.5	-3.6	3.7
2358	0	3.6	-2.9	-3.4	3.5	-3.4	3.5	3.5	-3.8	0	3.6	-3.0	-3.5	3.6	-3.5	3.5	3.5	-3.8
2367	25	3.6	-3.0	-3.5	3.4	3.4	-3.5	-3.5	3.8	36	3.6	-3.0	-3.5	3.5	3.5	-3.5	-3.6	3.8
2368	8	3.6	-2.9	-3.5	3.4	3.5	-3.4	3.5	-3.8	16	3.6	-2.9	-3.5	3.5	3.6	-3.4	3.6	-3.7

2378	25	3.6	-3.1	-3.4	3.5	3.5	3.6	-3.3	-3.8	36	3.6	-3.1	-3.5	3.5	3.5	3.6	-3.4	-3.8
2456	35	3.6	-2.9	3.4	-3.5	-3.5	-3.5	3.4	3.8	45	3.6	-2.9	3.5	-3.5	-3.6	-3.5	3.5	3.8
2457	26	3.6	-2.9	3.5	-3.5	-3.5	3.4	-3.5	3.8	34	3.6	-2.9	3.5	-3.5	-3.6	3.5	-3.5	3.7
2458	26	3.6	-3.0	3.5	-3.4	-3.3	3.6	3.5	-3.8	36	3.6	-3.0	3.5	-3.5	-3.4	3.6	3.6	-3.8
2467	2	3.6	-2.9	3.4	-3.5	3.4	-3.5	-3.5	3.8	10	3.6	-3.0	3.5	-3.5	3.5	-3.6	-3.5	3.8
2468	7	3.6	-2.9	3.4	-3.5	3.5	-3.4	3.6	-3.8	14	3.6	-2.9	3.5	-3.5	3.6	-3.4	3.6	-3.7
2478	7	3.6	-2.9	3.5	-3.4	3.5	3.5	-3.4	-3.8	17	3.6	-2.9	3.6	-3.4	3.5	3.5	-3.5	-3.8
2567	70	3.5	-3.0	3.3	3.4	-3.5	-3.3	-3.5	3.9	88	3.6	-3.0	3.4	3.4	-3.4	-3.3	-3.5	3.7
2568	56	3.6	-3.0	3.5	3.6	-3.4	-3.5	3.6	-3.8	54	3.7	-3.0	3.5	3.6	-3.5	-3.5	3.6	-3.8
2578	56	3.5	-3.2	3.5	3.6	-3.3	3.6	-3.4	-3.8	54	3.6	-3.2	3.5	3.6	-3.4	3.6	-3.5	-3.8
2678	64	3.7	-3.0	3.5	3.6	3.5	-3.5	-3.5	-3.8	64	3.7	-3.0	3.5	3.6	3.6	-3.5	-3.5	-3.8
3456	52	3.6	3.1	-3.3	-3.4	-3.1	-3.2	3.4	3.8	60	3.6	3.1	-3.3	-3.5	-3.1	-3.2	3.4	3.8
3467	43	3.6	3.1	-3.3	-3.3	3.4	-3.2	-3.1	3.8	49	3.6	3.1	-3.5	-3.3	3.4	-3.2	-3.1	3.8
3468	55	3.6	3.1	-3.3	-3.3	3.6	-3.0	3.6	-3.7	49	3.6	3.1	-3.3	-3.4	3.6	-3.1	3.6	-3.7
3567	62	3.5	3.0	-3.4	3.4	-3.2	-3.3	-3.3	4.0	82	3.7	3.1	-3.3	3.4	-3.2	-3.3	-3.3	3.7
3568	78	3.6	3.2	-3.1	3.5	-3.3	-3.2	3.6	-3.8									
3578	74	3.7	3.1	-3.3	3.6	-3.2	3.5	-3.1	-3.8	70	3.7	3.1	-3.3	3.6	-3.2	3.5	-3.0	-3.8
3678	91	3.7	3.2	-3.4	3.5	3.6	-3.2	-3.2	-3.8	91	3.7	3.2	-3.4	3.6	3.7	-3.2	-3.2	-3.8
4568	113	3.7	3.2	3.6	-3.4	-3.1	-3.2	3.6	-3.8	110	3.7	3.2	3.6	-3.4	-3.0	-3.2	3.6	-3.8
4578	98	3.7	3.1	3.5	-3.3	-2.9	3.5	-3.3	-3.8	91	3.7	3.1	3.6	-3.3	-2.9	3.6	-3.3	-3.8
5678	139	3.8	3.1	3.5	3.5	-2.9	-3.2	-3.4	-3.9	143	3.8	3.1	3.5	3.6	-3.0	-3.0	-3.5	-3.9

Table S22. Relative energies (ΔE in kJ/mol) and spin densities of the various BS states for the E_1 state of Fe-nitrogenase, calculated with B3LYP and TPSS with the large QM system using the Fe2 protonation state.

BS	B3LYP									TPSS								
	ΔE	Fe1	Fe2	Fe3	Fe4	Fe5	Fe6	Fe7	Fe8	ΔE	Fe1	Fe2	Fe3	Fe4	Fe5	Fe6	Fe7	Fe8
1234	96	-3.8	-3.1	-3.7	-3.6	3.7	3.7	3.6	3.8	151	-3.7	-2.6	-2.9	-3.0	3.0	3.0	3.2	3.8
1235	32	-3.5	-3.4	-3.5	3.8	-3.8	3.6	3.6	3.8									
1236	73	-3.7	-3.2	-3.6	3.7	3.6	-3.7	3.7	3.8	75	-3.1	-2.4	-2.4	2.8	2.5	-3.0	2.8	3.6
1237	92	-3.7	-3.4	-3.6	3.7	3.6	3.7	-3.6	3.8	87	-3.2	-2.0	-2.9	2.9	2.5	2.5	-3.0	3.7
1245	42	-3.7	-3.3	3.7	-3.6	-3.7	3.8	3.6	3.8	77	-3.3	-2.3	3.0	-2.8	-3.0	2.7	2.8	3.7
1246										72	-3.5	-2.7	2.7	-3.2	3.2	-2.7	3.2	3.7
1247	88	-3.6	-3.2	3.7	-3.7	3.6	3.7	-3.7	3.8									
1248										68	-3.3	-2.4	3.0	-2.5	3.2	3.2	3.2	-3.6
1256	55	-3.7	-3.2	3.6	3.7	-3.7	-3.6	3.7	3.8	27	-3.3	-2.5	3.1	3.1	-3.0	-3.0	2.9	3.6
1257	52	-3.7	-3.4	3.6	3.6	-3.6	3.8	-3.6	3.8	6	-3.3	-2.7	2.9	2.9	-2.8	3.0	-2.9	3.6
1258	66	-3.7	-3.3	3.6	3.7	-3.6	3.7	3.7	-3.8	43	-3.3	-2.5	3.2	3.1	-2.7	3.1	3.3	-3.6
1267	56	-3.7	-3.3	3.7	3.7	3.7	-3.6	-3.6	3.8	33	-3.3	-2.5	2.9	3.0	3.0	-3.0	-3.0	3.6
1268										45	-3.3	-2.6	3.2	3.1	3.3	-2.5	3.3	-3.6
1278	74	-3.7	-3.4	3.7	3.7	3.7	3.7	-3.6	-3.8	51	-3.3	-2.5	3.0	3.2	3.3	3.1	-2.6	-3.6
1345		-3.6	3.7	-3.5	-3.6	-3.7	3.6	3.8	3.8	77	-3.2	2.3	-2.9	-2.7	-2.9	3.1	3.1	3.7
1346	90	-3.6	3.4	-3.4	-3.5	3.7	-3.7	3.8	3.8	69	-3.2	2.4	-2.8	-2.9	3.2	-3.0	3.2	3.7
1348										75	-3.1	2.5	-1.7	-2.4	3.1	3.1	3.1	-3.6
1356	76	-3.6	3.3	-3.5	3.7	-3.6	-3.5	3.7	3.8	13	-3.2	2.3	-2.7	3.0	-2.8	-2.4	3.1	3.6
1357	96	-3.6	3.1	-3.4	3.7	-3.4	3.7	-3.3	3.7	18	-3.2	2.3	-2.7	3.1	-2.8	2.8	-2.1	3.5
1358	42	-3.6	3.2	-3.5	3.8	-3.5	3.7	3.8	-3.8	59	-3.1	2.5	-2.6	3.1	-2.1	3.3	3.2	-3.6
1367	83	-3.6	3.3	-3.6	3.8	3.7	-3.5	-3.5	3.8	37	-3.2	2.4	-2.8	3.0	2.9	-2.6	-2.5	3.6
1368										61	-3.3	2.6	-2.6	3.3	3.3	-2.3	3.1	-3.5
1378										51	-3.2	2.5	-2.2	3.1	3.3	3.3	-2.6	-3.6
1456	115	-3.6	3.3	3.7	-3.5	-3.5	-3.5	3.7	3.8	42	-3.2	2.5	3.0	-2.8	-2.1	-2.7	2.8	3.6
1457	100	-3.6	3.1	3.7	-3.4	-3.2	3.7	-3.5	3.8	23	-3.2	2.3	3.0	-2.7	-1.9	2.8	-3.0	3.6
1458										57	-3.2	2.5	3.2	-2.1	-2.5	3.3	3.3	-3.6
1467										9	-3.2	2.4	2.9	-2.9	3.1	-2.3	-2.8	3.6
1468	88	-3.6	3.4	3.7	-3.5	3.8	-3.4	3.7	-3.7	57	-3.2	2.6	3.3	-2.6	3.1	-2.2	3.3	-3.5
1478	40	-3.6	3.2	3.8	-3.5	3.8	3.8	-3.5	-3.7	62	-3.2	2.6	3.1	-2.6	3.2	3.3	-2.2	-3.6
1567	152	-3.8	3.0	3.7	3.7	-3.3	-3.3	-3.3	4.0	17	-3.2	2.2	2.9	2.9	-2.6	-2.2	-2.7	3.7
1568	44	-3.6	3.4	3.7	3.8	-3.5	-3.4	3.7	-3.8	30	-3.4	2.3	3.1	2.9	-2.8	-0.2	3.0	-3.6
1578	24	-3.6	3.2	3.8	3.8	-3.5	3.8	-3.5	-3.8	34	-3.3	2.4	3.0	3.1	-0.8	3.0	-2.6	-3.6
1678										33	-3.4	2.3	2.9	3.1	3.0	-0.3	-2.8	-3.6
2345	85	3.8	-3.0	-3.7	-3.6	-3.8	3.5	3.5	3.8	27	3.4	-2.3	-3.0	-3.1	-3.1	2.5	2.8	3.7
2346	8	3.5	-3.2	-3.6	-3.6	3.7	-3.7	3.7	3.8	28	3.2	-2.3	-2.9	-3.1	2.5	-3.1	2.7	3.7
2347	43	3.6	-3.1	-3.7	-3.6	3.7	3.7	-3.7	3.8	25	3.4	-2.3	-3.1	-3.0	2.7	2.4	-3.1	3.7
2348	104	3.8	-2.9	-3.6	-3.7	3.6	3.6	3.5	-3.7	21	3.2	-2.2	-2.5	-2.6	2.7	3.0	2.7	-3.6
2356	47	3.7	-3.1	-3.7	3.7	-3.6	-3.7	3.7	3.8	12	3.2	-2.4	-3.0	2.9	-3.1	-3.1	2.9	3.6
2357	50	3.7	-3.1	-3.7	3.7	-3.7	3.7	-3.7	3.8	18	3.2	-2.4	-2.9	3.0	-3.1	2.6	-3.2	3.6

2358	42	3.7	-3.1	-3.7	3.7	-3.7	3.7	3.6	-3.8	0	3.2	-2.4	-2.9	3.1	-2.9	3.0	3.0	-3.6
2367	55	3.7	-3.2	-3.7	3.6	3.7	-3.7	-3.6	3.8	35	3.2	-2.4	-3.0	2.7	2.8	-3.0	-3.2	3.6
2368	42	3.7	-3.1	-3.7	3.7	3.7	-3.6	3.7	-3.8	8	3.2	-2.3	-3.0	2.8	3.1	-2.6	3.1	-3.5
2378	43	3.7	-3.4	-3.6	3.7	3.7	3.7	-3.6	-3.8	34	3.2	-2.5	-2.9	3.0	3.1	3.1	-2.8	-3.6
2456	68	3.7	-3.1	3.7	-3.7	-3.6	-3.7	3.7	3.8	35	3.2	-2.3	2.7	-2.9	-3.1	-3.0	2.8	3.6
2457	59	3.7	-3.1	3.7	-3.7	-3.7	3.7	-3.7	3.8	21	3.2	-2.3	3.0	-3.0	-3.2	2.7	-3.1	3.6
2458	0	3.7	-3.3	3.7	-3.6	-3.7	3.8	3.7	-3.8	30	3.2	-2.3	3.0	-2.9	-2.8	3.1	3.1	-3.7
2467	40	3.7	-3.1	3.7	-3.7	3.7	-3.7	-3.6	3.8	7	3.2	-2.4	2.9	-3.0	3.0	-3.1	-3.2	3.6
2468	22	3.7	-3.1	3.7	-3.7	3.7	-3.6	3.7	-3.8	6	3.2	-2.3	2.8	-3.0	3.1	-2.6	3.2	-3.6
2478	53	3.7	-3.0	3.7	-3.7	3.6	3.7	-3.7	-3.8	1	3.2	-2.3	3.1	-2.9	3.0	3.0	-2.9	-3.6
2567	110	3.7	-3.3	3.7	3.7	-3.6	-3.6	-3.7	3.7	69	3.3	-2.4	2.7	2.8	-3.0	-3.0	-3.0	3.6
2568	61	3.7	-3.2	3.7	3.7	-3.7	-3.6	3.7	-3.8	88	3.4	-2.1	3.1	3.2	-3.0	-2.9	3.0	-3.7
2578	104	3.6	-3.4	3.7	3.7	-3.6	3.8	-3.6	-3.8	78	3.3	-2.4	3.1	3.2	-2.9	3.1	-2.9	-3.7
2678	19	3.7	-3.2	3.7	3.7	3.7	-3.7	-3.7	-3.8	88	3.5	-2.2	3.2	3.1	2.9	-3.0	-3.0	-3.7
3456	107	3.7	3.3	-3.5	-3.6	-3.3	-3.5	3.6	3.8	27	3.2	2.5	-2.8	-3.0	-2.7	-2.7	2.9	3.6
3467	97	3.7	3.3	-3.6	-3.5	3.6	-3.5	-3.3	3.8	20	3.3	2.5	-3.0	-2.9	2.9	-2.7	-2.7	3.6
3468	26	3.7	3.4	-3.5	-3.6	3.8	-3.5	3.8	-3.8	34	3.2	2.6	-2.5	-2.7	3.1	-2.5	3.1	-3.5
3567										55	3.3	2.5	-2.9	2.9	-2.6	-2.8	-3.0	3.6
3568										75	3.4	2.7	-2.4	3.1	-2.8	-2.5	3.1	-3.7
3578										70	3.5	2.7	-2.7	3.3	-2.9	2.9	-2.3	-3.7
3678										95	3.5	2.8	-2.8	3.2	3.0	-2.6	-2.8	-3.7
4568										110	3.5	2.8	3.2	-2.6	-2.3	-2.8	2.9	-3.7
4578	126	3.7	3.2	3.7	-3.5	-3.2	3.7	-3.5	-3.8	99	3.4	2.6	3.1	-2.5	-2.1	3.0	-3.0	-3.7
5678										155	3.7	2.8	3.1	3.2	-2.3	-2.8	-2.9	-3.8
